# Integration of TGF-β-induced Smad signaling in the insulin-induced transcriptional response in endothelial cells

**DOI:** 10.1038/s41598-019-53490-x

**Published:** 2019-11-18

**Authors:** Erine H. Budi, Steven Hoffman, Shaojian Gao, Ying E. Zhang, Rik Derynck

**Affiliations:** 1 Departments of Cell and Tissue Biology, and Anatomy, Eli and Edythe Broad Center of Regeneration Medicine and Stem Cell Research, University of California at San Francisco, San Francisco, CA 94143-0669 USA; 2 Thoracic and Gastrointestinal Oncology Branch, Center for Cancer Research, National Cancer Institute, Bethesda, MD 20892-1906 USA; 3 Laboratory of Cellular and Molecular Biology, Center for Cancer Research, National Cancer Institute, Bethesda, MD 20892-4256 USA

**Keywords:** Growth factor signalling, Transcriptomics

## Abstract

Insulin signaling governs many processes including glucose homeostasis and metabolism, and is therapeutically used to treat hyperglycemia in diabetes. We demonstrated that insulin-induced Akt activation enhances the sensitivity to TGF-β by directing an increase in cell surface TGF-β receptors from a pool of intracellular TGF-β receptors. Consequently, increased autocrine TGF-β signaling in response to insulin participates in insulin-induced angiogenic responses of endothelial cells. With TGF-β signaling controlling many cell responses, including differentiation and extracellular matrix deposition, and pathologically promoting fibrosis and cancer cell dissemination, we addressed to which extent autocrine TGF-β signaling participates in insulin-induced gene responses of human endothelial cells. Transcriptome analyses of the insulin response, in the absence or presence of a TGF-β receptor kinase inhibitor, revealed substantial positive and negative contributions of autocrine TGF-β signaling in insulin-responsive gene responses. Furthermore, insulin-induced responses of many genes depended on or resulted from autocrine TGF-β signaling. Our analyses also highlight extensive contributions of autocrine TGF-β signaling to basal gene expression in the absence of insulin, and identified many novel TGF-β-responsive genes. This data resource may aid in the appreciation of the roles of autocrine TGF-β signaling in normal physiological responses to insulin, and implications of therapeutic insulin usage.

## Introduction

Cell responses to extracellular ligands often lead to changes in gene expression, resulting from receptor-mediated activation of signaling pathways. These then target genes through post-translational modifications and changes in chromatin binding of DNA-binding transcription factors. Accordingly, extracellular ligands, such as growth factors, activate or repress sets of genes that are identified as cognate target genes, responsive to the ligand. The extent to which target genes are regulated often depends on signaling crosstalk or cooperation of the ligand-induced signaling pathway with other pathways, and are therefore thought of as positive or negative modifiers of ligand-induced transcription responses.

Similar to many growth factors, insulin, a hormone that is secreted by cells in the pancreas, controls the expression of a variety of insulin-responsive genes that in turn control many essential functions at the cellular level and regulate glucose uptake and metabolism^1,2^. Insulin action is initiated by its interaction with a cognate cell surface tyrosine kinase receptor complex, leading to activation of kinase pathways, most notably the PI3K-Akt-mTOR and Erk MAPK signaling pathways^3^. Downstream from ligand-receptor binding and induction of signaling are target genes that, in response to insulin, are activated or repressed, and therefore are seen as insulin-responsive^4^. A number of insulin target genes have been identified and their mechanism of activation has been studied. On a larger scale, genome-wide studies provide better insight into the gene expression responses that are exerted in response to insulin, and these have been done in many different cells including hepatoma cells^4–6^, mouse fibroblasts^7,8^, osteoclasts precursors^9^, human skeletal muscle^10^, placenta^11^ and endothelial cells^12,13^. Although many studies have looked into the effect of insulin in these cells, most large scale analyses did not take into account the participation or integration of other signaling pathways that may contribute to insulin-induced transcriptional responses.

We reported that Akt activation in response to insulin induces a rapid increase in cell surface TGF-β receptors in fibroblasts, epithelial cells, and endothelial cells, through mobilization of TGF-β receptors from an intracellular pool to the cell surface^13,14^. Increased cell surface presentation of TGF-β receptors confers increased sensitivity to TGF-β, thus enhancing autocrine TGF-β signaling responses, raising the possibility that the insulin-induced increase in autocrine TGF-β signaling participates in gene expression and cellular responses to insulin. Indeed, we showed that elevated autocrine TGF-β signaling in response to insulin contributed to insulin-induced angiogenic responses in cell culture and *ex vivo*^13^. These findings invite questions regarding the extent to which TGF-β signaling participates and integrates in the response to insulin. For example, what biological processes, functions and pathways are controlled by insulin through enhanced TGF-β signaling or with contributions of TGF-β/Smad signaling?

To address the extent to which insulin-induced upregulation of autocrine TGF-β signaling contributes to insulin-induced gene expression, we carried out genome-wide RNA-Seq analyses of human umbilical vein endothelial cells (HuVECs). Endothelial cells are a prime target of insulin^15–17^; they are exposed to insulin in the circulation, and mediate insulin transport from circulation into skeletal muscle^1,18^. Additionally, endothelial cells play central roles in the control of vascular homeostasis and integrity, inflammatory responses, and pathogenesis of many diseases including cardiovascular diseases, diabetes, fibrosis, and cancer^1,19–22^. We performed paired RNA-Seq studies on HUVECs treated or not with insulin in the presence or absence of the TGF-β receptor kinase inhibitor, SB431542, that blocks TGF-β -induced Smad activation, thus the TGF-β /Smad-mediated transcription responses^23,24^. Our data revealed that both insulin and TGF-β signaling inhibition affect the expression of a large number of genes associated with various biological processes and signaling pathways. Venn analyses of the differentially expressed genes identified numerous genes that integrate TGF-β/Smad signaling into the insulin-induced response, and these genes associate with a variety of biological responses and signaling pathways. Consequently, the genome-wide transcription changes in response to insulin are substantially restricted when TGF-β signaling is prevented, indicating crosstalk and participation of TGF-β signaling in insulin transcriptional responses. Finally, we demonstrate that basal gene expression of endothelial cells in the absence of insulin depends on autocrine TGF-β signaling.

## Results

### Concentration-dependent, insulin-induced Smad2 and Smad3 activation in endothelial cells

We previously reported that insulin increases TGF-β responsiveness and autocrine TGF-β /Smad signaling by promoting the transport of TGF-β receptors to the cell surface, and that this stimulation of TGF-β receptor transport results from insulin-induced Akt activation^14^. We also reported that the increased autocrine TGF-β response contributes to the insulin-induced angiogenic response^13^. To better appreciate the extent to which increased autocrine TGF-β contributes to insulin-induced changes in gene expression, we performed a genome-wide survey of changes in gene expression in response to insulin in human umbilical cord endothelial cells HUVECs, and assessed the contribution of TGF-β-induced Smad activation to the transcriptomic response.

**Figure 1:**
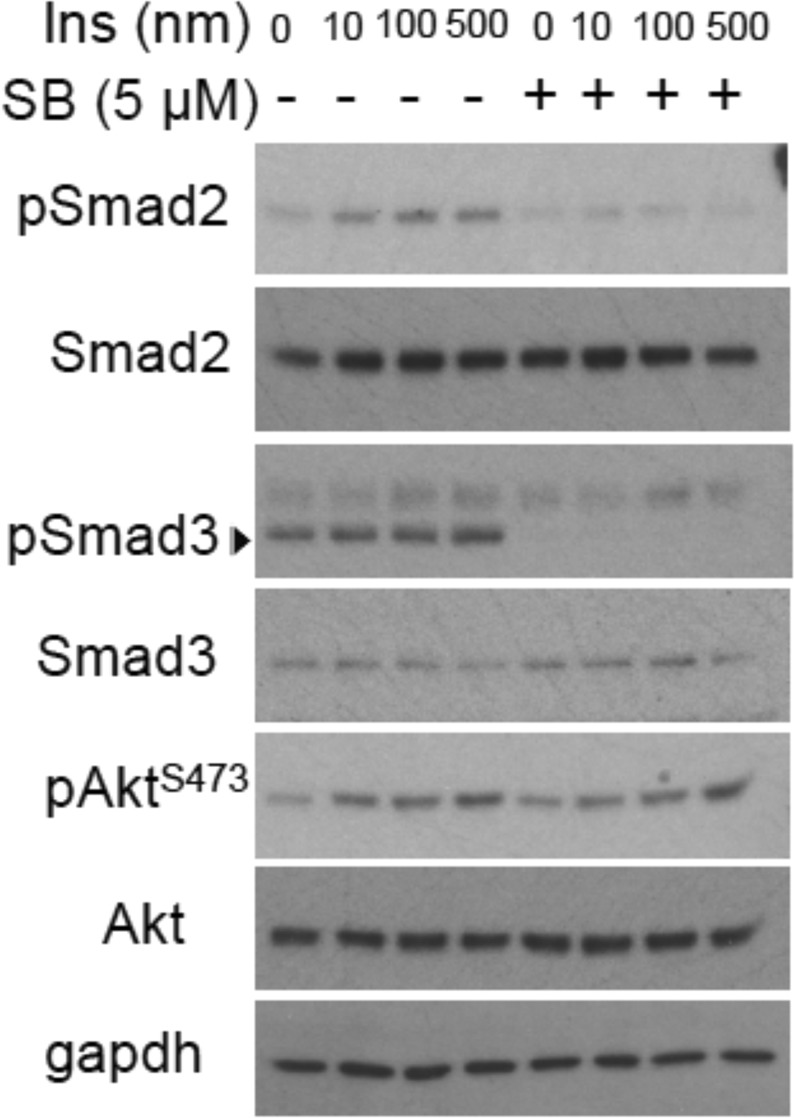
Dose response of insulin-induced Smad2 and Smad3 activation. HUVECs were treated with insulin (Ins) in the presence or absence of SB431542 for 30 min using the indicated concentrations. Smad2 and Smad3 activation were assessed by immunoblotting for C-terminally phosphorylated Smad2 (pSmad2) or Smad3 (pSmad3). Insulin-induced Akt activation was assessed by immunoblotting for phosphorylated Akt (pAkt^S473^). GAPDH was used as loading control. The samples from the same experiment were run on 2 gels that were processed in parallel.Original blots are presented in Supplementary Fig. 1.

Insulin-induced gene expression changes depend on the concentration and duration of insulin exposure^4,12^. We therefore first evaluated the extent to which autocrine TGF-β signaling is activated in response to insulin. HUVECs were treated for 30 min with increasing concentrations of insulin. The TGF-β activation were assessed by examining the Smad2 and Smad3 activation levels by immunoblotting for C-terminally phosphorylated Smad2 or Smad3. As shown in Fig. 1 (Supplemental Fig S1 for original blots), Smad2 and Smad3 activation levels increased, as we increased the insulin concentration. The levels of phospho-Smad2 showed maximal increase at 100 nM insulin, while Smad3 activation increased in a concentration-dependent manner up to 500 nM insulin. Akt phosphorylation at Ser473 also increased in a concentration-dependent manner up to 500 nM of insulin. SB431542, an inhibitor of the type I TGF-β receptor TβRI, prevented activation of Smad2 and Smad3, but did not affect insulin-induced Akt activation. Similar to HUVECs, increasing concentration of insulin treatment of human microvascular lung endothelial cells (HMVEC-L) induced increased Smad2 and Smad3 activation and addition of SB431542 prevented the response (Supplemental Fig. S2 and S3 for original blots).

Based on this result, we elected to perform our transcriptome analyses using cells stimulated by insulin at 100 nM, i.e. the concentration that was also used to evaluate the contribution of autocrine TGF-β signaling to insulin-induced angiogenic response of endothelial cells^13^.

### Genome-wide transcription responses to insulin and autocrine TGF-β signaling

**Figure 2:**
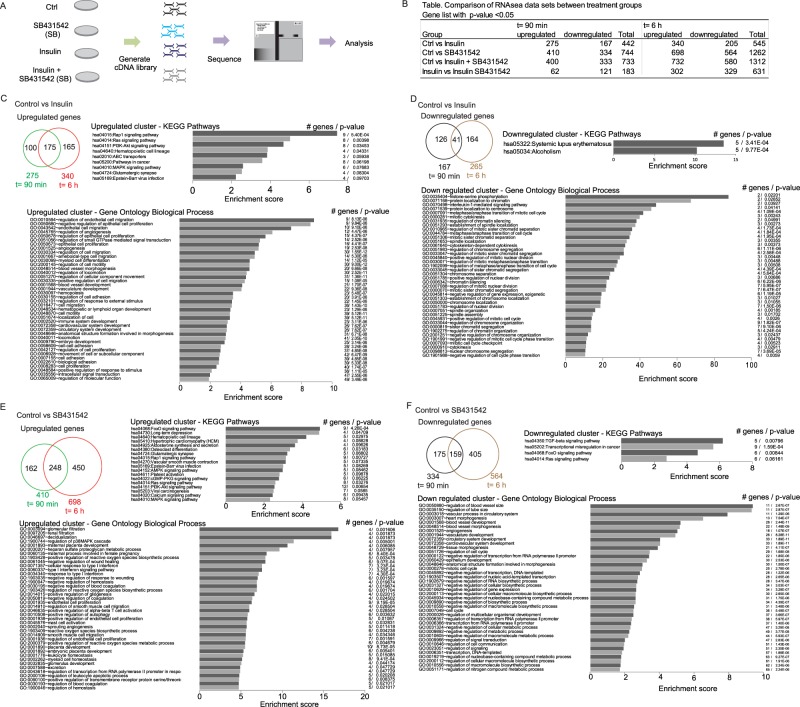
RNA sequencing (RNA-Seq) of HUVECs treated or not with insulin in the presence or absence of the TGF-β signaling inhibitor SB431542. (**A**) Scheme of the RNA-Seq experimental design. mRNA collected from HUVECs treated with 100 nM insulin in the presence or absence of 5 µM SB431542 for 90 min or 6 h were reverse transcribed and cDNA libraries were generated that were then subjected to sequencing and analysis. (**B**) Summary of RNA-Seq differential expression analyses at 90 min and 6 h after initiation of treatment. Gene expression changes with an absolute fold change ≥ 1.27 or log 0.35 and p < 0.05 were considered significant. (**C–F**) Venn analyses (top left) showing the distribution of differentially expressed genes that were upregulated or downregulated in response to insulin (**C,D**) or SB431542 (E, F) after 90 min (t = 90 min) and 6 h (t = 6 h) of treatment. KEGG pathway analyses and top 40 gene ontology process enrichment groups associated with genes that showed increased or decreased expression at both 90 min and 6 h treatment, Number of genes and p-values are shown on the right side of the graph.

To define the contribution of the insulin-induced increase in autocrine TGF-β/Smad signaling to the insulin-induced transcriptional response in endothelial cells, we treated cells with or without insulin for 90 min or 6 h in the absence or presence of the TβRI kinase inhibitor SB431542. Treatment of the cells with SB431542 in the absence of insulin allowed us to evaluate the contribution of autocrine TGF-β signaling to gene expression under basal conditions, and served as control for cells treated with insulin in the presence of SB431542 (Fig. 2A). RNA-Seq data were collected using paired-end sequencing of three samples each for insulin (ins), SB431542 (SB) and insulin + SB431542 (ins + SB), and two samples each for controls at 90 min and 6 h. Gene expression that was enhanced or repressed at two different time points was defined on the basis of absolute fold change of > (0.35 log2) and p ≤ 0.05 (Fig. 2B). The RNA-Seq data obtained from insulin-, SB431542- and insulin + SB431542-treated cells were compared with data from control cells, and data from cells treated with insulin were compared with those from cells treated with insulin + SB431542 (Fig. 2B). The full lists of genes that met the criteria are provided in Supplemental Table 1 for 90 min treatment, and Supplemental Table 2 for 6 h treatment.

Based on our cut-off criteria, we identified 442 and 545 insulin-responsive genes at 90 min or 6 h, respectively. Conversely, SB431542 treatment identified 744 and 1262 genes whose expression levels under basal cell culture conditions were enhanced or repressed by autocrine TGF-β signaling. The majority of genes that were responsive to insulin or SB431542 responded by increasing their expression (Fig. 2B). Overall, the number of genes identified as regulated genes was higher after 6 h of treatment, when compared to 90 min treatment. Of the 275 genes that were upregulated after 90 min insulin treatment, 64% (175/275) continued to be upregulated after 6 h, while 24% (41/167) of the insulin-responsive genes that were downregulated at 90 min stayed repressed after 6 h (Fig. 2C,D, Supplemental Table 3). Among those genes that were upregulated after 90 min in response to SB431542, 60% (248/410) continued to be upregulated after 6 h, while 48% (159/334) of the genes that were downregulated at 90 min stayed repressed after 6 h (Fig. 2E,F, Supplemental Table 3).

We functionally annotated the 175 genes whose expression was enhanced at both 90 min and 6 h in response to insulin using the DAVID bioinformatics tool. Our results revealed enrichment of pathways known to associate with insulin, such as Ras, PI3K-Akt, ABC transporter, pathways in cancer, and MAPK signaling, as well as pathways that are less known to associate with the insulin response, such as Rap1 signaling, cell migration, proliferation, adhesion, angiogenesis, blood vessel development, hematopoiesis, immune system development, and response to stimulus (Fig. 2C, Supplemental Table 4). Enrichment analysis on the 41 genes that were repressed in response to insulin at both 90 min and 6 h revealed association with lupus erythematosus and alcoholism, and cell cycle and chromatin remodeling processes (Fig. 2D, Supplemental Table 5).

Enrichment analysis of the 248 genes that were continuously upregulated in response to SB431542, and therefore were repressed by autocrine TGF-β signaling, revealed many pathways including FoxO, osteoclast-related, aldosterone-related, Rap1, AMPK, cGMP-PKG, PI3K-Akt, calcium signaling, and MAPK signaling (Fig. 2E, Supplemental Table 6). 520 gene ontology (GO) terms linked to the 248 upregulated genes, with the top 40 GO terms including kidney filtration, regulation of heparin sulfate metabolic processes, repression of wound healing, interferon signaling, promotion of endothelial proliferation and autophagy, T cell activation, blood vessel sprouting, and placental development (Fig. 2E, Supplemental Table 6). Functional annotation of the159 genes that were continuously repressed in response to SB431542, and therefore were stimulated by autocrine TGF-β signaling, revealed TGF-β pathway signaling (as expected), transcriptional misregulation in cancer, and FoxO and Ras signaling, while gene ontology terms linked to blood vessel development, negative regulation of RNA, gene expression, cellular metabolic processes and cell cycle (Fig. 2F, Supplemental Table 7).

### Increased autocrine TGF-β responsiveness contributes extensively to the insulin-induced transcriptome changes

**Figure 3:**
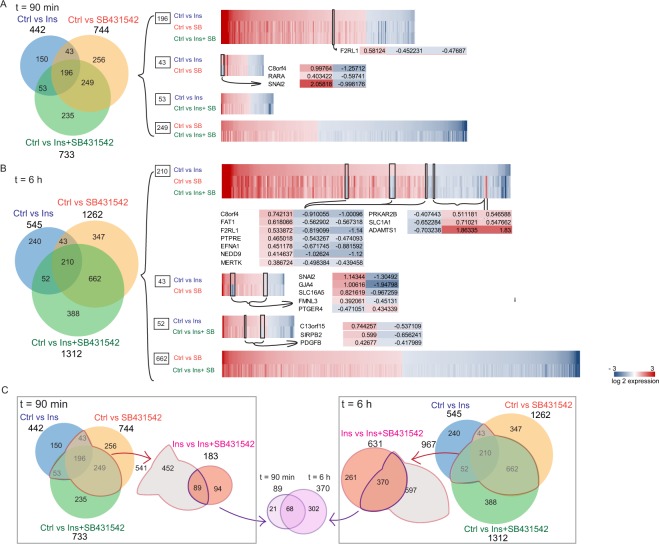
Venn and cluster analyses of differentially expressed genes in HUVECs treated with insulin (Ins), SB431542 or insulin + SB431542 (Ins + SB431542), in comparison to control. (**A,B**) Venn diagram showing the distribution of differentially expressed genes ( > 0.35 log2 in expression and p ≤ 0.05) at 90 min or 6 h, comparing Ins versus SB431542 and/or Ins + SB431542 treatments. Clustered heatmaps to the right show differentially expressed genes that are shared between three or two comparison groups. Red color shows upregulated genes while blue color indicates downregulated gene expression compared to control. Values shown are log2-based. Differentially expressed genes with opposing expression trends when comparing treatments are shown below or besides the heatmap. (**C**) Comparisons between 90 min and 6 h treatment groups. The pools of shared genes among 541 differentially expressed genes at 90 min (C, left) and 967 genes after 6 h (C, right) were compared with those that overlapped between Ins and Ins + SB431542 treatments at the times shown. Further comparison of genes that are shared with Ins versus Ins + SB431542 at the two time points are shown in purple in the center.

To gain insight into the contribution of autocrine signaling to the genome-wide gene expression changes in response to insulin, we performed RNA-Seq-based Venn analyses of differentially expressed genes at 90 min and at 6 hours of treatment (Fig. 3A–C).

Among the 442 genes that were induced or repressed in response to insulin at 90 min, 239 genes or 54% had their expression affected by the presence of SB431542. The 442 insulin-responsive genes also showed a 56% (249/442 genes) overlap with those that are enhanced or repressed in response to insulin + SB431542, while 60% (445/744) of the SB431542-responsive genes were shared with those whose expression is changed in response to insulin + SB431542 (Fig. 3A, Supplemental Table 8). Evaluating the differentially expressed genes that are shared by the three comparison groups identified 196 genes whose expression is affected by insulin, SB431542, as well as insulin + SB431542. We note that, of the 442 insulin-responsive genes, only 150 (34%) were not affected by SB431542, and, thus, did not have a contribution of autocrine TGF-β signaling (Fig. 3A, Supplemental Table 8).

Similar analyses of regulated genes after 6 h of treatment led to similar conclusions, but involved a larger number of differentially regulated genes and larger amplitudes in their regulation (Fig. 3B). In these analyses, the expression of 545 genes was enhanced or repressed by insulin, and 253 (46%) of these were also controlled by autocrine TGF-β signaling, i.e. were affected by SB431542. 240 (44%) out of the 545 genes were not affected by autocrine TGF-β signaling, and 210 genes (38%) had their expression regulated by insulin, SB431542 and insulin + SB431542 (Fig. 3B, Supplemental Table 9). Together, the Venn analyses at 90 min and 6 h of treatment revealed an extensive overlap between genes that are responsive to insulin and those that are controlled by autocrine TGF-β signaling. These results reveal significant participation of autocrine TGF-β signaling in insulin-induced gene responses.

Heat maps of the differentially expressed genes after 90 min and 6 h treatment provided further insight into integration of autocrine TGF-β signaling in insulin-induced gene expression changes. For most genes, blocking TGF-β signaling using SB431542 affected the amplitude of insulin-induced gene activation or repression, without fully reversing the response. However, several genes that were induced in response to insulin were repressed when TGF-β signaling was prevented by SB431542, and a few genes saw insulin-induced repression reverted to activation in response to SB431542 (Fig. 3A,B, Supplemental Tables 8 and 9). Notably, in the 196-gene cluster that is shared by the three comparison groups at 90 min of treatment, the *F2RL1* gene that encodes the proteinase-activated receptor 2 (PAR2)^25^, and three of the 43 genes that are shared between insulin- and SB431542-regulated genes, i.e. *c8orf4*, *RARA* and *SNAI2*, had their basal and insulin-induced expression repressed by SB431542. *SNAI2* gene encodes a master transcription factor that drives epithelial- and endothelial-to-mesenchymal transition^26^. *c8orf4* encodes Thyroid Cancer Protein-1 (TCP-1), which functions as positive regulator in the Wnt/b-catenin signaling pathway^27^, and *RARA* encodes the retinoic acid receptor-α, which controls processes in development, differentiation, apoptosis and granulopoiesis^28,29^ (Fig. 3A). Among the genes identified at 6 h of treatment, additional genes showed reversal of their insulin-induced activation or repression when autocrine TGF-β signaling was blocked (Fig. 3B). Among those regulated by insulin, SB431542 and insulin + SB431542, seven genes, including *c8orf4* and *F2RL1* that were already induced at 90 min, and *FAT1, PTPRE, EFNA1, NEDD9*, and *MERTK* were induced by insulin but inhibited by SB431542 or insulin + SB431542, and three genes were downregulated by insulin and upregulated by SB431542 and insulin + SB431542. Of the 43 genes that were shared by the insulin- and SB431542-reponsive gene groups at 6 h, five showed reversal of the insulin response by SB431542. *SNAI2*, *GJA4* (encoding a gap junction protein)^30^, *SLC16A5* (encoding a monocarbohydrate transporter)^31^ and *FMNL3* (formin-like 3)^32^ were upregulated in response to insulin but repressed by SB431542, whereas the *PTGER4* gene, encoding a prostaglandin E2 receptor^33^, was inhibited by insulin and induced by SB431542. Among the 52 differentially expressed genes that were shared by the insulin and insulin + SB431542 groups, three showed opposing expression patterns. These were *c13orf15*, which encodes a cell cycle regulator^34,35^, *SIRPB2* encoding signal regulatory protein β2^36^ and *PDGFB*, which encodes a well-known growth factor and angiogenic mediator^37,38^ (Fig. 3B). As apparent from these analyses of heat maps, increased autocrine TGF-β signaling in response to insulin helps define the insulin-induced activation or repression of a very substantial fraction of insulin-responsive genes, and fully mediates the insulin-induced response of a select set of genes.

Finally, comparing cells treated with insulin with those treated with insulin + SB431542 identified 183 and 631 genes with differential expression at 90 min and 6 h, respectively (Fig. 2B, Supplemental Tables 1 and 2); thus, autocrine TGF-β signaling contributed to their insulin response at 90 min and 6 h after adding insulin. As shown in Fig. 3C (left) and Supplemental Table 10, 89 of these 183 genes identified at 90 min, were among the overlapping genes identified in Fig. 3A. Similarly, of the 631 genes identified by comparing insulin and insulin + SB431542 treatments at 6 h, 370 were also among the overlapping genes identified in Fig. 3B (Fig. 3C, right, Supplemental Table 10). Of these 89 and 370 genes, 68 were genes that were shared by those at the two time points (Fig. 3C, Supplemental Table 10).

### Functional annotation of TGF-β-dependent insulin target genes

**Figure 4:**
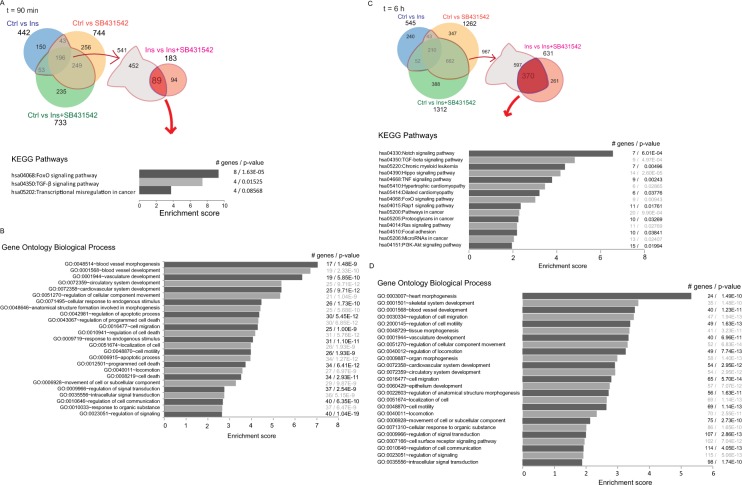
Functional annotation of differentially expressed genes after 90 min and 6 h treatment with insulin (Ins), SB431542 or insulin + SB431542 (Ins + SB431542). Enrichment analysis of the 89 genes at 90 min (**A,B**) and 370 genes at 6 h (**C,D**) analyzed using the DAVID functional annotation program. KEGG pathway (**A,C**) and the top 25 GO biological process terms (**B,D**) associated with the shared differentially expressed genes are shown. The number of genes and p-values associated with each term are shown on the right side.

We next aimed to gain further insights into the functional roles of those genes that were up- or downregulated in response to insulin, dependent on autocrine TGF-β signaling. For this purpose we focused on both the 89 and 370 genes identified after 90 min or 6 hours insulin treatment (Fig. 4A–D, Supplemental Table 11), and performed Gene Ontology (GO) and pathway analysis based on the KEGG database. Functional pathway annotation using the DAVID bioinformatics tool of the common 89 differentially genes at 90 min suggested three pathways, i.e. FoxO and TGF-β signaling, and transcription misregulation in cancer (Fig. 4A, Supplemental Table 12). Ontology analysis of these genes resulted in 772 categories related to various biological responses (Supplemental Table 12). Most notably, the top 25 functional biological process categories, based on the p-value, related to blood vessel morphogenesis and development, apoptosis, cell migration and movement, as well as regulation of signal transduction (Fig. 4B, Supplemental Table 12).

Analyzing the 370 genes identified after 6 h treatment using DAVID bioinformatics software identified 22 enriched pathways based on the KEGG database, and 1270 gene ontology terms for biological responses (Fig. 4C,D, Supplemental Table 13). These included FoxO and TGF-β signaling that were already identified at 90 min, and now again enriched at 6 h. As expected and reported for insulin^39^, also Ras signaling and PI3K-Akt pathway genes were enriched. Additional pathways included Notch, Hippo, TNF and Rap1 signaling, as well as cancer-associated regulation pathways (Fig. 4C, Supplemental Table 13). The top 25 functional biological process categories, based on the p-value, continued to relate to morphogenesis of the heart and skeletal system, blood vessel and vasculature development, cell migration and movement, as well as regulation of signal transduction (Fig. 4D, Supplemental Table 13). These results were consistent with the annotated pathways for differentially expressed, insulin-responsive genes at 90 min.

**Figure 5:**
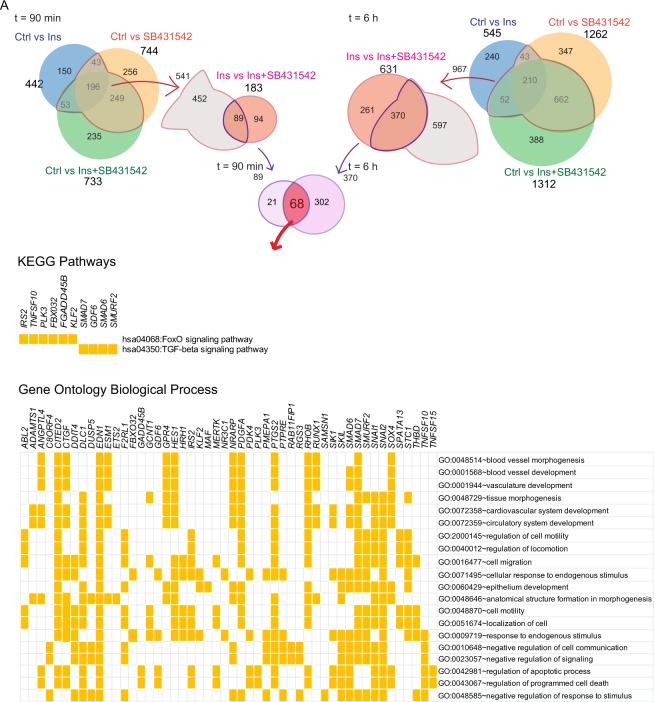
KEGG pathway and gene ontology biological process enrichment analysis of 68 diferentially expressed genes that are shared between the 90 min and 6 h treatment groups. The KEGG pathways and top 20 gene ontology biological process terms with associated genes are listed in the panel.

DAVID functional gene annotation analysis based on the 68 genes that are shared by the 90 min and 6 h treatments highlighted the importance of the FoxO and TGF-β signaling pathways and revealed 657 gene ontology term categories for biological processes (Fig. 5, Supplemental Table 14). The top 20 ontology processes (Fig. 5, bottom), were consistent with those at 90 min and 6 h (Fig. 4). Together, these analyses further support the notion that increased autocrine TGF-β signaling in response to insulin integrates into insulin-induced gene expression responses in blood vessel development, tissue morphogenesis, cardiovascular development, cell movement, apoptosis and programmed cell death, as well as cellular metabolic processes.

### Confirmation of RNA-Seq results using qRT-PCR

**Figure 6:**
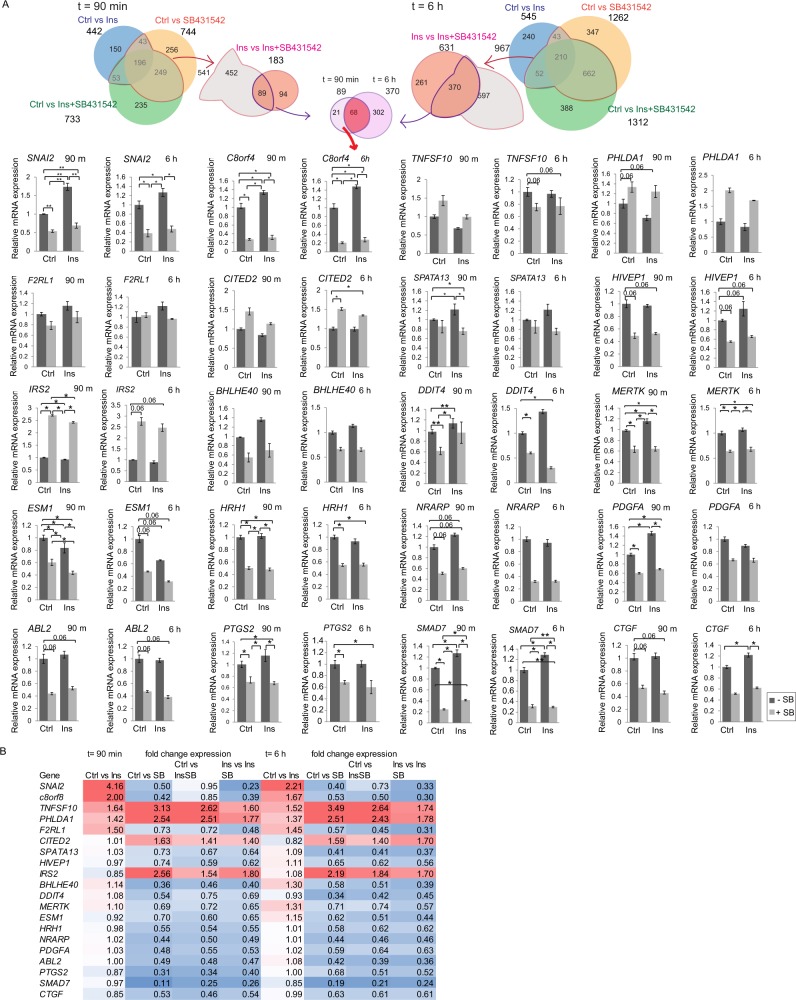
Validation of RNA-Seq data by qRT-PCR. (**A**) Relative mRNA levels of selected genes that are shared between 90 min and 6 h groups are shown. HUVECs were treated with or without 100 nM insulin in the presence or absence of 5 μM SB431542 for 90 min or 6 h. mRNA expression of the indicated genes after 90 min and 6 h treatment was measured using qRT-PCR, and values were normalized to RPL13 mRNA. The statistical significance was determined by Wilcoxon test. Error bars indicate standard error of the means, based on three independent experiments. *p < 0.05, **p < 0.0083 (**B**) RNA-Seq data on the fold expression changes of genes compared to control or insulin treatment. Red color indicates an increase and blue color indicates a decrease in fold change gene expression compared to control.

To validate the RNA-Seq results, we selected 20 differentially regulated genes among the TGF-β-dependent, insulin-regulated genes (Fig. 6) and evaluated the effects of insulin in the presence or absence of autocrine TGF-β signaling using qRT-PCR analysis (Fig. 6A). Their expression patterns correlated well to those seen using RNA-Seq, although quantitative differences between qRT-PCRT and RNA-Seq results were apparent, especially with those that showed low RNA-Seq fold changes, as expected. For example, qRT-PCR analysis showed that insulin treatment induced expression of *SNAI2*, *c8orf4*, *F2RL1*, *SPATA13*, *HIVEP1*, *BHLHE40*, *MERTK*, and that blocking autocrine TGF-β signaling with SB431542 substantially reduced the expression of most genes, including *SNAI2*, *c8orf4*, *F2RL1*, *SPATA13*, *HIVEP1*, *BHLHE40*, *DDIT4*, *MERTK*, *ESM*, *HRH1*, *NRARP*, *PDGFA*, *ABL2*, *PTGS2*, *SMAD7* and *CTGF* (Fig. 6A). In contrast, autocrine TGF-β signaling dampened the responses of some genes, e.g. *PHLDA1*, *IRS2* and *ESM1*. These qRT-PCR data are mostly consistent with the RNA-Seq findings, showing similar patterns for insulin-induced changes and effects of SB431542 inhibition (Fig. 6B, Supplemental Table 15). Blocking TGF-β signaling using the pan-TGF-β neutralizing 1D11 antibody, which prevents ligand binding to its receptors, confirmed the participation of autocrine TGF-β signaling in insulin-induced expression changes of select target genes, similar to addition of SB431542 (Supplemental Fig. S4). Among the 20 genes comparing insulin treatment with control, the direction of fold change of *TNFSF10*, *PHLDA1* and *ESM* differed between RNA-Seq and the qRT-PCR. Increasing or decreasing the concentration of insulin or SB431542 showed dose-dependent changes in the mRNA levels (Supplemental Fig. S5). The effects of insulin in HMVEC-L cells in the presence or absence of autocrine TGF-β signaling, i.e. using SB431542 or 1D11 antibody to neutralize the ligand (Supplemental Figs. S6 and S7), were similar to the effects of insulin and SB431542 on gene expression in HUVECs (Fig. 6).

### Regulatory gene sequences of TGF-β-dependent insulin target genes

Our RNA-Seq results revealed a large variety of genes that respond to both TGF-β and insulin, and illustrated the integration of autocrine TGF-β signaling in the insulin transcriptomic responses. We therefore anticipated to locate cis-regulatory sequence motifs known to respond to insulin and TGF-β/Smad signaling. Insulin signaling has been linked to target gene activation or repression through binding of transcription factors on insulin response elements (IREs). At least eight distinct consensus insulin response sequences (IRSs) have been identified^40–45^.

**Figure 7:**
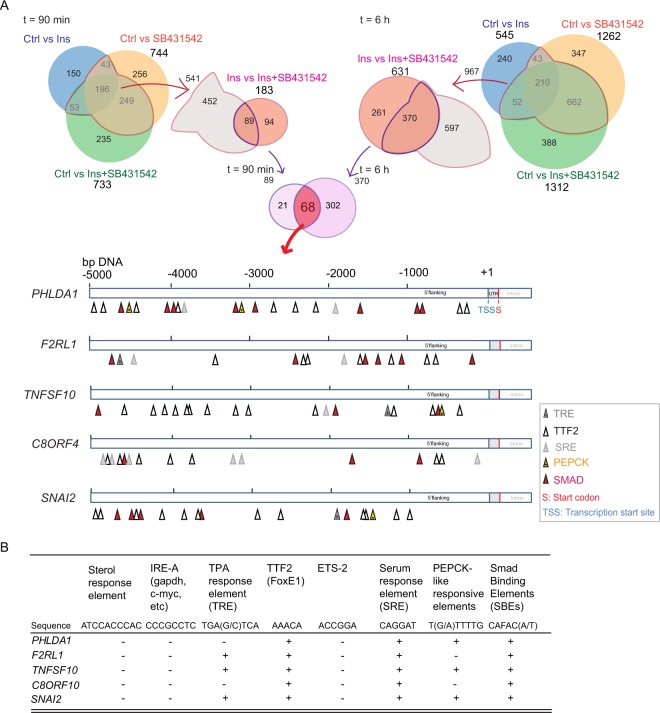
Identification of insulin-responsive and Smad3-binding elements within the 5 kbp sequences proximal to the transcription start site. (**A**) Illustration showing predicted locations of cis-elements that confer insulin or TGF-β/Smad3 responsiveness. Colored triangles mark response elements known to be regulated by insulin or Smad3. (**B**) Summary table of insulin-responsive and Smad-binding elements. Plus marks indicate the presence of at least one element.

Scanning the 5 kbp DNA proximal to the transcription start site of five insulin- and TGF-β-responsive genes that were identified using RNA-Seq, i.e. *PHLDA1*, *F2RL1*, *TNFSF10*, *C8ORF4*, and *SNAI2*, for published sequence motifs revealed known insulin response elements and Smad binding motifs (Fig. 7A). Accordingly, these five genes showed, by qRT-PCR, to have insulin-responsive expression that is modulated by autocrine TGF-β signaling (Fig. 6). Specifically, they all contained TGF-β -responsive Smad binding elements (SBEs), as well as serum response elements (SREs) and TTF2 binding sites, which were shown to confer insulin responsiveness. The insulin-responsive PEPCK binding site was found in the *PHLDA1*, *TNFSF10*, and *SNAI2* regulatory sequences but not in *F2RL1* and *C8ORF4*, while a TPA-response element that also has been linked to insulin responsiveness was observed in *F2RL1*, *TNFSF10* and *SNAI2*. Thus, each of the five genes showed that at least two insulin response elements and one Smad binding sequence (Fig. 7B). We also tested the effects of TGF on a subset of the genes shown in Fig. 6 and saw the activation or inhibition effect of TGF-β (Suppl. S8). These analyses further support and may explain our findings that these genes respond to insulin and integrate TGF-β signaling in their response.

### RNA-Seq profiling reveals novel early TGF-β target genes

In addition to insights in the contributions of TGF-β signaling to the insulin-induced genomic response, our RNAseq results at 90 min allowed us to identify novel early target genes of the TGF-β/Smad pathway, i.e. as opposed to secondary gene activation that may result from Smad-mediated expression of transcription factors. Indeed, comparing gene expression in the absence versus presence of SB431542 identified many genes that are repressed or activated by SB431542 at 90 min (Fig. 2, Supplemental Table 1, and Suplemental Table 2). Among these, we focused on genes that were transcriptionally repressed by SB431542 at 90 min.

**Figure 8:**
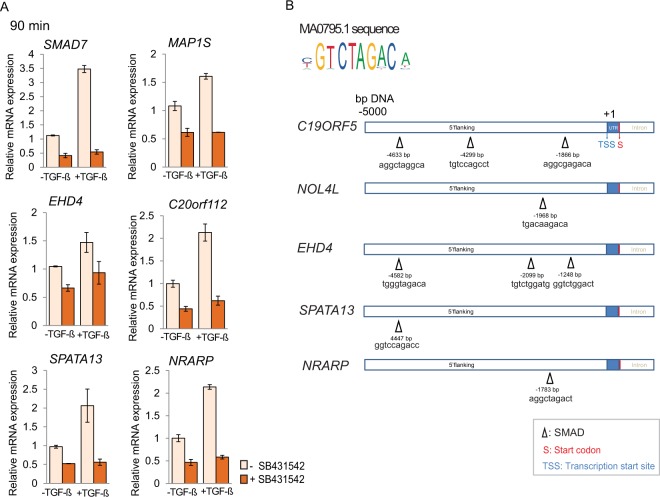
Analysis of potentially direct TGF-β/Smad3 target genes. (**A**) qRT-PCR of mRNA extracted from HUVECs treated with or without TGF-β in the presence or absence of SB431542 for 90 min, normalized against RPL13 mRNA. (**B**) Using the consensus sequence available from JASPAR database, the 5 kbp DNA sequences upstream from the transcription start of each gene were scanned for Smad3-binding motifs. Potential Smad binding sites are marked by triangles.

We tested five early Smad/TGF-β-dependent genes that were not known to be TGF-β responsive, i.e. *MAP1S*, *EHD4*, *c20orf112*, *SPATA13* and *NRARP* (Fig. 8A), and compared their responses with *SMAD7*, an established direct TGF-β /Smad-responsive gene (Fig. 8A). *MAP1S*, originally identified as *c19orf5*, encodes a widely expressed microtubule-associated protein that promotes autophagy, and, at increased expression, associates with poor prognosis of ovarian cancer^46–48^. *EHD4*, which stands for Eps15 Homology Domain 4 encodes an endocytic regulatory protein that has not been studied much^49,50^. *C20orf112* encodes nucleolar protein 4-like (NOL4L), a largely uncharacterized protein found in the cytoplasm and nucleus^51^. The protein encoded by *SPATA13* associates with cytoskeletal remodeling during migration, adhesion assembly and disassembly^52,53^. *NRARP*, which stands for Notch-regulated ankyrin repeat protein, acts downstream of Notch signaling, and functions in cancer progression, cancer stem cell self-renewal, angiogenesis, as well as in patterning, and development^54–57^.

Since direct TGF-β/Smad target genes have regulatory DNA sequences that recruit Smad complexes, we performed an in silico search for Smad3-binding elements (SBE) motifs using binding profiles from the JASPAR CORE database of experimentally defined transcription factor binding sites for Smad3^58^. A score is calculated for the probed sequence that provides a measure of similarity to the Smad3 transcription factor consensus sequence. Supporting our RNAseq data and PCR results, we found consensus Smad3 transcription factor binding sites, i.e. the GTCTAGAC sequence, in the flanking regions of all five early TGF-β target genes; their presence was experimentally confirmed through PCR analysis (Fig. 8B). Our in silico analysis of transcription factor binding sites for Smad3 is consistent with their direct regulation by TGF-β/Smad signaling.

## Discussion

This study provides a genome-wide characterization of the effects of insulin on gene expression and, more specifically, the extensive contribution and integration of autocrine TGF-β signaling in the gene expression responses of human endothelial cells to insulin. Our results also highlight the largely unappreciated role of autocrine TGF-β signaling in basal gene expression in cell culture. We hope that our results will serve as a resource for further studies not only on the response to insulin, but also on the control of cell physiology by autocrine TGF-β signaling.

To evaluate the role of autocrine TGF-β signaling in the insulin response, we treated cells with insulin for 90 min to examine early responses that are likely to not depend on new protein synthesis, and for 6 hours to attain more durable gene responses and incorporate somewhat later gene responses, which may or may not depend on new protein synthesis. We treated endothelial cells with insulin at 100 nM, which induces Smad2 and Smad3 activation as a result of increased autocrine TGF-β signaling (Fig. 1), and is commonly used to study effects of insulin in cell culture^59–62^. We note that at a 10-fold lower concentration, insulin also induces Smad2 and Smad3 activation, albeit at a lower level (Fig. 1).

Our genome-wide RNA-Seq analysis identified hundreds of insulin-responsive genes at 90 min and 6 hours of treatment. At 90 min, 62% (275 out of 442) genes were upregulated and 38% (167 out of 442) were downregulated in response to insulin, and similar percentages of upregulated and downregulated genes were seen after 6 hours of insulin. The higher proportion of genes that is upregulated is consistent with the anabolic role of insulin, and a microarray study of gene expression changes in skeletal muscle after a 3-hour hyperinsulenemic clamp^63^. The insulin-induced gene expression changes in our study suggest regulation of various KEGG-based pathways, including Rap1, Ras, PI3K-Akt and MAPK signaling as well as ABC transporter function, consistent with a hepatoma and liver RNA-Seq study^6,64^ and other insulin studies^3,65–67^. Our GO annotation related mainly to endothelial cell migration, proliferation, angiogenesis, immune system as well as cardiovascular system development, consistent with the functional nature of endothelial cells. Insulin is known to promote carbohydrate and lipid metabolism^17^. Although our functional annotation showed enrichment for these metabolic processes, they were not among the top 20 hits in our insulin GO terms list. Further examination of the enrichment results comparing DEGs between the 90 min and the 6 h insulin treated cells indicated more genes associated with these carbohydrate and lipid-related metabolic processes after 6 h of insulin treatment. Supporting our data, it has been reported in rat H4IIE hepatoma cells that insulin-induced effects of genes related to glycolysis, fatty acid oxidation and lipid metabolism are less distinct at 6 h when compared to 24 h insulin exposure^5^.

Central in the current study is the integration of autocrine TGF-β signaling into insulin-induced gene expression changes, which was predicted from our observation that insulin-induced Akt activation promotes rapid mobilization of intracellular receptors to the cell surface, resulting in increased TGF-β responsiveness autocrine TGF-β signaling^14^. To assess this integration, endothelial cells were treated with insulin in the absence or presence of SB431542 to block TGF-β/Smad signaling. We found that about half of the insulin-responsive genes showed decreased or increased expression when autocrine TGF-β/Smad signaling was prevented. These results reveal that, in response to insulin, many genes are coregulated by insulin and TGF-β signaling and that TGF-β signaling extensively participates in the insulin response. Many of the genes that were coregulated at both 90 min and 6 h of insulin treatment associated with FoxO and TGF-β signaling pathways, and biological processes involved in blood vessel development, cardiovascular system, cell migration, apoptosis, and cell communication. These associations are consistent with our observations that elevated autocrine TGF-β signaling in response to insulin contributes to insulin-induced angiogenic responses^13^.

The regulation of insulin-responsive gene expression through cooperation of autocrine TGF-β/Smad signaling with insulin receptor signaling predicts the presence of both insulin-responsive and Smad3-binding sequences in regulatory promoter sequences of target genes. We performed in silico analyses of 5 kbp sequences preceding the transcription start sites of several coordinately regulated, insulin-responsive genes, i.e. *PHLDA1*, *F2RL1*, *TNFSF10*, *C8orf4* and *SNAI2*. Previous research identified eight insulin-responsive sequences in regulatory regions of insulin target genes^40–45^, whereas direct TGF-β/Smad responsiveness results from binding of Smad3/4 complexes to CAGA-like Smad binding elements^68,69^. Each of the insulin-responsive, coregulated genes examined contained at least two insulin response elements, as well as Smad3 binding elements, thus providing a molecular basis for the coregulation.

The insulin-induced enhancement of autocrine TGF-β signaling also predicts that insulin induces TGF-β/Smad target genes whose regulation does not directly emanate from the activated insulin receptor. This is indeed the case. Among the insulin-responsive genes in our analyses, we recognize genes that are known as direct TGF-β/Smad3 target genes. Additionally, insulin-induced activation or repression of a number of genes is fully prevented when TGF-β signaling is blocked, indicating that insulin induces these through enhanced autocrine TGF-β signaling.

In the current study, we also present a genome-wide analysis of the effect of autocrine TGF-β signaling inhibition on gene expression in untreated, control endothelial cells. Various studies report on effects of TGF-β on genome-wide transcription, comparing results of maximal TGF-β stimulation against those of either untreated cells or cells treated to inhibit TGF-β signaling. These studies in different cell types revealed gene expression changes of relevance to a variety of cellular processes and functions, including cellular homeostasis, growth and proliferation, and cell and tissue differentiation, including epithelial-mesenchymal transition of carcinoma cells^70–77^. Our current results complement and support these previous studies, but, more importantly, provide to our knowledge the first genome-wide analysis of the role of autocrine TGF-β signaling in directing the basal transcription program of cells in endothelial cells. Our analyses comparing cells treated with SB431542 to inhibit TGF-β/Smad-signaling with untreated control endothelial cells shows that basal autocrine TGF-β activity controls various KEGG pathways, including FoxO, Ras, Rap1, AMPK, cGMP-PKG, PI3K-Akt, calcium and MAPK signaling. These pathways overlap with the non-Smad TGF-β signaling response^78^, which can be activated through autocrine signaling^79^. Functional ontology analyses suggest roles of autocrine TGF-β signaling in many processes, including blood vessel development, gene transcription, cell metabolism, interferon signaling, blood function and activation, as well as basic cell functions such as cell migration, autophagy, and proliferation. These basic processes are involved in epithelial to mesenchymal transition, a differentiation process in normal development, wound healing and pathogenesis of cancer and fibrosis^80–82^. Our findings on the role of autocrine TGF-β signaling in insulin-induced angiogenic responses support the notion that basal TGF-β signaling also participates in various other processes.

Finally, our data also reveal novel direct target genes for TGF-β/Smad signaling. While TGF-β regulate the expression of many genes, the number of early direct TGF-β/Smad target genes has been limited to only few, which imposes restrictions on reliable marker profiles to evaluate efficacy of therapeutic inhibition of TGF-β in immuno-oncology and anti-fibrotic approaches. Among newly identified TGF-β target genes, we identify *MAP1S*, *EHD4*, *c20orf112*, *SPATA13* and *NRARP* as novel direct targets of TGF-β/Smad3 signaling. These genes are rapidly activated in response to TGF-β, and contain Smad3-binding sites upstream from their transcription initiation sites. Further exploration of our data bases may therefore not only serve those who are interested in the cellular response to insulin, but also be of great interest to those who aspire to identify TGF-β responsive genes.

In summary, we show that the genome-wide changes in mRNA expression in response to insulin integrate substantial contributions of autocrine TGF-β signaling. This mode of signaling crosstalk needs to be highlighted not only when evaluating the cell responses to insulin, but also when trying to appreciate the full consequences of therapeutic use of insulin.

## Methods

### Cell culture

Human umbilical vein endothelial cells (HUVECs) were obtained from Gibco and cultured in Medium 200 (Invitrogen) with 10% fetal bovine serum (FBS) from HyClone, and low serum growth supplement (LSGS) from Invitrogen. Lung microvascular endothelial cells (HMVEC-L) were obtained from PromoCell. Cells were grown in endothelial cell growth medium MV2 (PromoCell) with supplements added. All cells were maintained in a humidified 5% CO2 incubator at 37 °C, and the medium was replaced every 2 days until the cells reached 80–90% confluence. All experiments were carried out using cells between 3rd and 7th passage.

Insulin from Sigma was used at 100 nM. The TβRI kinase inhibitor SB431542 from Sigma was used at 5 μM and added 45 min before treatment. The anti-TGF-β1, -β2, -β3 neutralizing antibody 1D11 (R&D Systems, MAB1835-SP) was used at 2 μg/ml. TGF-β from Humanzyme was used at 0.4 ng/ml.

### Immunoblotting

Cell lysis, protein extraction, and immunoblotting were performed as described^83^. Protein concentrations were quantified using Bradford assays (Bio-Rad) with bovine serum albumin (BSA) as standard, and a SpectraMax M5 microplate reader. Samples were resolved by SDS-PAGE on 4–12% gradient polyacrylamide gel. Proteins were transferred to a nitrocellulose membrane and detected by immunoblotting. As primary antibodies, we used rabbit monoclonal antibodies to Smad2, phospho-Smad2 (Ser^465/467^), Smad3, Akt, and phospho-Akt (p-Akt^S473^) from Cell Signaling. Antibody against phospho-Smad3 (Ser^423/425^) was from Abcam, and glyceraldehyde-3-phosphate dehydrogenase (GAPDH) was from Sigma. For immunoblotting, immunoreactive bands were visualized using Western Lighting Plus ECL (Perkin Elmer).

### cDNA library preparation for RNA-Seq

HUVECs were seeded onto six well plates at a density of 2 × 10^5^ per well and cultured as described above. At the second day, cells were starved overnight in Medium 200 without growth factors. The cells were treated with 100 nM insulin for 90 min or 6 h, with or without 5 μM SB431542. Total RNA was extracted from 22 independent samples of HUVECs, that is control 90 min (2 samples), control 6 h (2 samples), insulin 90 min (3 samples), insulin 6 h (3 samples), SB431542 90 min (3 samples), SB431542 6 h (3 samples), insulin + SB431542 90 min (3 samples), and insulin + SB431542 90 min (3 samples). The quality of total RNA after extraction using Qiagen RNAeasy Plus mini kit was checked by NanoDrop spectrophotometric readings at 260/280/230 nm and with an Agilent 2100 bioanalyzer. cDNA libraries were made using Encore Complete RNA-seq Library Systems kit from NUGEN following the manufacturer protocol. The quality of the library was checked using DNA1000. All 22 samples of barcoded library, four controls from the two time points and three of each of treated samples from the two time points, were sequenced in seven lanes using a paired-end 100 protocol on the Illumina HiSeq. 2000 at the UCSF Genomics Core Facilities.

### RNA-Seq differential expression analysis

Paired-end RNAseq sequencing reads were aligned with TopHat version 2.0.13^84^ (http://tophat.cbcb.umd.edu) against the reference human genome build hg19, with UCSC known gene transcripts as the gene model annotation. Transcripts were assembled using Cufflinks version 2.2.1, and differentially expressed genes were identified with Cuffdiff version 2.19 using an adjusted P-value of 0.05 or less based on the Benjamini and Hochberg correction^85,86^ (http://cufflinks.cbcb.umd.edu/). The demultiplxed RNA-Seq data reported in this paper were deposited in ArrayExpress with accession number E-MTAB-8297.

### Venn analyses

To visualize insections of genes that are differentially expressed by different groups, we used a free available online tool Venny 2.1^87^.

### Functional annotation

Functional and pathway analyses were performed mainly using the Database for Annotation, Visualization, and Integrated Discovery (DAVID, available at: https://david.ncifcrf.gov/)^88,89^. To understand the significance of genes, we performed Gene Ontology (GO) classification, making use of BP_Fat (biological process). We also used the Kyoto Encyclopedia of Genes and Genomes (KEGG) pathway enrichment analysis option in DAVID to detect the potential pathway of target genes.

### Validation using qRT-PCR

Complementary DNA was synthesized using an iScript cDNA synthesis Kit (Bio-Rad), according to manufacturer’s protocol. Complementary DNA samples were subjected to real-time PCR using Bio-Rad PCR and the double-stranded-specific dye SYBR green. For all primer pairs, specific amplification of the PCR products was confirmed by melting curve analysis. Primer sequences were designed using Primer3^90,91^ or taken from the PrimerBank database^92,93^, and are presented in Supplemental Table 16.

### Analysis of Smad3 binding elements

Smad3 transcription factor binding sites were predicted by the software JASPAR^58^. http://jaspar.genereg.net/search?q=Homo%20sapiens&collection=CORE&tax_group=vertebrates with Matrix ID: MA0795.1 was used to analyze the Smad3 potential binding elements. Submitted sequences of 5 kbp nucleotides upstream of the 5′UTR were analyzed using a relative profile score default threshold setting of 80% to the “CORE Vertebrate”database. Searches of 5′ flanking sequences were performed using Ensambl human database^94^. Insulin responsive elements were analyzed manually.

## Supplementary information

Supplemental Figures

Dataset 1

Dataset 2

Dataset 3

Dataset 4

Dataset 5

Dataset 6

Dataset 7

Dataset 8

Dataset 9

Dataset 10

Dataset 11

Dataset 12

Dataset 13

Dataset 14

Dataset 15

Dataset 16

## References

[1] Escudero CA (2017). Pro-angiogenic role of insulin: from physiology to pathology. Front Physiol.

[2] Tokarz VL, MacDonald PE, Klip A (2018). The cell biology of systemic insulin function. J Cell Biol.

[3] Taniguchi CM, Emanuelli B, Kahn CR (2006). Critical nodes in signalling pathways: insights into insulin action. Nat Rev Mol Cell Biol.

[4] Sano T (2016). Selective control of up-regulated and down-regulated genes by temporal patterns and doses of insulin. Sci Signal.

[5] Hectors TL, Vanparys C, Pereira-Fernandes A, Knapen D, Blust R (2012). Mechanistic evaluation of the insulin response in H4IIE hepatoma cells: new endpoints for toxicity testing?. Toxicol Lett.

[6] Kawata K (2018). Trans-omic analysis reveals selective responses to induced and basal insulin across signaling, transcriptional, and metabolic networks. iScience.

[7] Dupont J (2001). Insulin and IGF-1 induce different patterns of gene expression in mouse fibroblast NIH-3T3 cells: identification by cDNA microarray analysis. Endocrinology.

[8] Versteyhe S (2013). IGF-I, IGF-II, and insulin stimulate different gene expression responses through binding to the IGF-I receptor. Front Endocrinol.

[9] Kim HS, Lee NK (2014). Gene expression profiling in osteoclast precursors by insulin using microarray analysis. Mol Cells.

[10] Rome S (2003). Microarray profiling of human skeletal muscle reveals that insulin regulates approximately 800 genes during a hyperinsulinemic clamp. J Biol Chem..

[11] Lassance L (2015). Identification of early transcriptome signatures in placenta exposed to insulin and obesity. Am J Obstet Gynecol..

[12] Di Camillo B (2010). The transcriptional response in human umbilical vein endothelial cells exposed to insulin: a dynamic gene expression approach. PloS one.

[13] Budi EH, Mamai O, Hoffman S, Akhurst RJ, Derynck R (2019). Enhanced TGF-β; signaling contributes to the insulin-induced angiogenic responses of endothelial cells. iScience.

[14] Budi EH, Muthusamy BP, Derynck R (2015). The insulin response integrates increased TGF-β signaling through Akt-induced enhancement of cell surface delivery of TGF-β receptors. Sci Signal.

[15] King GL, Johnson SM (1985). Receptor-mediated transport of insulin across endothelial cells. Science.

[16] Jialal I (1985). Characterization of the receptors for insulin and the insulin-like growth factors on micro- and macrovascular tissues. Endocrinology.

[17] Barrett EJ, Liu Z (2013). The endothelial cell: an “early responder” in the development of insulin resistance. Rev Endocr Metab Disord.

[18] Chiu JD (2008). Direct administration of insulin into skeletal muscle reveals that the transport of insulin across the capillary endothelium limits the time course of insulin to activate glucose disposal. Diabetes.

[19] Gimbrone MA, Garcia-Cardena G (2016). Endothelial cell dysfunction and the pathobiology of atherosclerosis. Cir Res.

[20] Potenta S, Zeisberg E, Kalluri R (2008). The role of endothelial-to-mesenchymal transition in cancer progression. Br J Cancer.

[21] Piera-Velazquez S, Li Z, Jimenez SA (2011). Role of endothelial-mesenchymal transition (EndoMT) in the pathogenesis of fibrotic disorders. Am J Pathol.

[22] Kolka CM, Bergman RN (2013). The endothelium in diabetes: its role in insulin access and diabetic complications. Rev Endocr Metab Disord.

[23] Callahan JF (2002). Identification of novel inhibitors of the transforming growth factor β1 (TGF-β1) type 1 receptor (ALK5). J Med Chem.

[24] Laping NJ (2002). Inhibition of transforming growth factor (TGF)-β1-induced extracellular matrix with a novel inhibitor of the TGF-β type I receptor kinase activity: SB-431542. Mol Pharmacol.

[25] Adams MN (2011). Structure, function and pathophysiology of protease activated receptors. Pharmacol Ther.

[26] Lamouille S, Xu J, Derynck R (2014). Molecular mechanisms of epithelial-mesenchymal transition. Nat Rev Mol Cell Biol.

[27] Jung Y (2006). TC1 (C8orf4) enhances the Wnt/β-catenin pathway by relieving antagonistic activity of Chibby. Cancer Res.

[28] Kastner P (2001). Positive and negative regulation of granulopoiesis by endogenous RARalpha. Blood.

[29] Das BC (2014). Retinoic acid signaling pathways in development and diseases. Bioorg Med Chem.

[30] Inai T, Shibata Y (2009). Heterogeneous expression of endothelial connexin (Cx) 37, Cx40, and Cx43 in rat large veins. Anat Sci Int.

[31] Murakami Y (2005). Functional characterization of human monocarboxylate transporter 6 (SLC16A5). Drug Metab. Dispos.

[32] Katoh M (2003). Identification and characterization of human FMNL1, FMNL2 and FMNL3 genes in silico. Int J Oncol.

[33] Narumiya S (1997). Molecular diversity of prostanoid receptors; subtypes and isoforms of prostaglandin E receptor. Adv Exp Med Biol.

[34] Saigusa K (2007). RGC32, a novel p53-inducible gene, is located on centrosomes during mitosis and results in G2/M arrest. Oncogene.

[35] Huang WY (2009). RGC-32 mediates transforming growth factor-β-induced epithelial-mesenchymal transition in human renal proximal tubular cells. J Biol Chem.

[36] Barclay AN, Brown MH (2006). The SIRP family of receptors and immune regulation. Nat Rev Immunol.

[37] French WJ, Creemers EE, Tallquist MD (2008). Platelet-derived growth factor receptors direct vascular development independent of vascular smooth muscle cell function. Mol Cell Biol.

[38] Heldin CH (2013). Targeting the PDGF signaling pathway in tumor treatment. Cell Commun Signal.

[39] Boucher, J., Kleinridders, A. & Kahn, C. R. Insulin receptor signaling in normal and insulin-resistant states. *Cold Spring Harb Perspect Biol*, **6**, 10.1101/cshperspect.a009191 (2014). 10.1101/cshperspect.a009191 PMC3941218 24384568

[40] Perrone L, Pasca di Magliano M, Zannini M, Di Lauro R (2000). The thyroid transcription factor 2 (TTF-2) is a promoter-specific DNA-binding independent transcriptional repressor. Biochem Biophys Res Commun.

[41] O’Brien RM (1995). Hepatic nuclear factor 3- and hormone-regulated expression of the phosphoenolpyruvate carboxykinase and insulin-like growth factor-binding protein 1 genes. Mol Cell Biol.

[42] Framson P, Bornstein P (1993). A serum response element and a binding site for NF-Y mediate the serum response of the human thrombospondin 1 gene. J Biol Chem.

[43] Fernandez-Alvarez A, Soledad Alvarez M, Cucarella C, Casado M (2010). Characterization of the human insulin-induced gene 2 (INSIG2) promoter: the role of Ets-binding motifs. J Biol Chem.

[44] Philippe J (1991). Insulin regulation of the glucagon gene is mediated by an insulin-responsive DNA element. Proc Natl Acad Sci USA.

[45] Sutherland C, O. B. R., Granner DK. (Landes Bioscience, Austin (TX), 2000–2013).

[46] Xie R (2011). Microtubule-associated protein 1S (MAP1S) bridges autophagic components with microtubules and mitochondria to affect autophagosomal biogenesis and degradation. J Biol Chem.

[47] Song K (2015). Transforming growth factor TGFβ increases levels of microtubule-associated protein MAP1S and autophagy flux in pancreatic ductal adenocarcinomas. PloS One.

[48] Orban-Nemeth Z, Simader H, Badurek S, Trancikova A, Propst F (2005). Microtubule-associated protein 1S, a short and ubiquitously expressed member of the microtubule-associated protein 1 family. J Biol Chem.

[49] Kuo HJ, Tran NT, Clary SA, Morris NP, Glanville RW (2001). Characterization of EHD4, an EH domain-containing protein expressed in the extracellular matrix. J Biol Chem.

[50] Sharma M, Naslavsky N, Caplan S (2008). A role for EHD4 in the regulation of early endosomal transport. Traffic.

[51] Guastadisegni MC (2010). CBFA2T2 and C20orf112: two novel fusion partners of RUNX1 in acute myeloid leukemia. Leukemia.

[52] Jean L (2014). The Rho family GEF Asef2 regulates cell migration in three dimensional (3D) collagen matrices through myosin II. Cell adhes migr.

[53] Kawasaki Y (2007). Identification and characterization of Asef2, a guanine-nucleotide exchange factor specific for Rac1 and Cdc42. Oncogene.

[54] Ishitani T, Matsumoto K, Chitnis AB, Itoh M (2005). Nrarp functions to modulate neural-crest-cell differentiation by regulating LEF1 protein stability. Nat Cell Biol.

[55] Lamar E (2001). Nrarp is a novel intracellular component of the Notch signaling pathway. Genes Dev.

[56] Phng LK (2009). Nrarp coordinates endothelial Notch and Wnt signaling to control vessel density in angiogenesis. Dev Cell.

[57] Zhang M (2017). Overexpression of NOTCH-regulated Ankyrin Repeat Protein is associated with papillary thyroid carcinoma progression. PloS one.

[58] Khan A (2018). JASPAR 2018: update of the open-access database of transcription factor binding profiles and its web framework. Nucleic Acids Res.

[59] Jaldin-Fincati JR, Pereira RVS, Bilan PJ, Klip A (2018). Insulin uptake and action in microvascular endothelial cells of lymphatic and blood origin. Am J Physiol Endocrinol Metab.

[60] Mammi C (2011). Sildenafil reduces insulin-resistance in human endothelial cells. PloS one.

[61] Montagnani M (2002). Inhibition of phosphatidylinositol 3-kinase enhances mitogenic actions of insulin in endothelial cells. J Biol Chem.

[62] Meng D (2012). NADPH oxidase 4 mediates insulin-stimulated HIF-1α and VEGF expression, and angiogenesis *in vitro*. PloS One.

[63] Rudkowska I, Jacques H, Weisnagel SJ, Marette A, Vohl MC (2013). Transcriptomic profiles of skeletal muscle tissue following an euglycemic-hyperinsulinemic clamp in insulin-resistant obese subjects. Genes Nutr.

[64] Fukuchi M (2001). Ligand-dependent degradation of Smad3 by a ubiquitin ligase complex of ROC1 and associated proteins. Mol Biol Cell.

[65] Okada S, Matsuda M, Anafi M, Pawson T, Pessin JE (1998). Insulin regulates the dynamic balance between Ras and Rap1 signaling by coordinating the assembly states of the Grb2-SOS and CrkII-C3G complexes. EMBO J.

[66] Nonomura K, Arai Y, Mitani H, Abe-Dohmae S, Yokoyama S (2011). Insulin down-regulates specific activity of ATP-binding cassette transporter A1 for high density lipoprotein biogenesis through its specific phosphorylation. Atherosclerosis.

[67] Toyoki D (2017). Insulin stimulates uric acid reabsorption via regulating urate transporter 1 and ATP-binding cassette subfamily G member 2. Am J Physiol Renal Physiol.

[68] Dennler S (1998). Direct binding of Smad3 and Smad4 to critical TGF β-inducible elements in the promoter of human plasminogen activator inhibitor-type 1 gene. Embo j.

[69] Zawel L (1998). Human Smad3 and Smad4 are sequence-specific transcription activators. Mol Cell.

[70] Horiguchi K (2012). TGF-β drives epithelial-mesenchymal transition through δEF1-mediated downregulation of ESRP. Oncogene.

[71] Li Y (2014). RNA-Seq and network analysis revealed interacting pathways in TGF-β-treated lung cancer cell lines. Cancer Inform.

[72] Kirwan RP, Leonard MO, Murphy M, Clark AF, O’Brien CJ (2005). Transforming growth factor-β-regulated gene transcription and protein expression in human GFAP-negative lamina cribrosa cells. Glia.

[73] Renzoni EA (2004). Gene expression profiling reveals novel TGF-β targets in adult lung fibroblasts. Respir Res.

[74] Foroutan M, Cursons J, Hediyeh-Zadeh S, Thompson EW, Davis MJ (2017). A Transcriptional program for detecting TGFβ-Induced EMT in cancer. Mol Cancer Res.

[75] Ranganathan P (2007). Expression profiling of genes regulated by TGF-β: differential regulation in normal and tumour cells. BMC genomics.

[76] Sundar R, Gudey SK, Heldin CH, Landström M (2015). TRAF6 promotes TGFβ-induced invasion and cell-cycle regulation via Lys63-linked polyubiquitination of Lys178 in TGFβ type I receptor. Cell Cycle.

[77] Xie L (2003). Transforming growth factor β-regulated gene expression in a mouse mammary gland epithelial cell line. Breast Cancer Res.

[78] Zhang, Y. E. Non-Smad signaling pathways of the TGF-β Family. *Cold Spring Harb Perspect Biol*, **9**, 10.1101/cshperspect.a022129 (2017). 10.1101/cshperspect.a022129 PMC5287080 27864313

[79] Duan D, Derynck R (2019). Transforming growth factor β (TGF-β)-induced up-regulation of TGF-β receptors at the cell surface amplifies the TGF-β response. J Biol Chem.

[80] Dongre A, Weinberg RA (2019). New insights into the mechanisms of epithelial-mesenchymal transition and implications for cancer. Nat Rev Mol Cell Biol.

[81] Kim, K. K., Sheppard, D. & Chapman, H. A. TGF-β1 signaling and tissue fibrosis. *Cold Spring Harb Perspect Biol*, **10**, 10.1101/cshperspect.a022293 (2018). 10.1101/cshperspect.a022293 PMC5880172 28432134

[82] Colella, B., Faienza, F. & Di Bartolomeo, S. EMT regulation by autophagy: a new perspective in glioblastoma biology. *Cancers*, **11**, 10.3390/cancers11030312 (2019). 10.3390/cancers11030312 PMC6468412 30845654

[83] Wu L, Derynck R (2009). Essential role of TGF-β signaling in glucose-induced cell hypertrophy. Dev Cell.

[84] Kim D (2013). TopHat2: accurate alignment of transcriptomes in the presence of insertions, deletions and gene fusions. Genome Biol.

[85] Trapnell C (2013). Differential analysis of gene regulation at transcript resolution with RNA-seq. Nat Biotechnol.

[86] Trapnell C (2010). Transcript assembly and quantification by RNA-Seq reveals unannotated transcripts and isoform switching during cell differentiation. Nat Biotechnol.

[87] Oliveros, J. C. V. An interactive tool for comparing lists with Venn’s diagrams. http://bioinfogp.cnb.csic.es/tools/venny/index.html (2007–2015).

[88] Huang DW, Sherman BT, Lempicki RA (2009). Systematic and integrative analysis of large gene lists using DAVID bioinformatics resources. Nat Protoc.

[89] Huang da W, Sherman BT, Lempicki RA (2009). Bioinformatics enrichment tools: paths toward the comprehensive functional analysis of large gene lists. Nucleic Acids Res.

[90] Koressaar T, Remm M (2007). Enhancements and modifications of primer design program Primer3. Bioinformatics.

[91] Untergasser A (2012). Primer3–new capabilities and interfaces. Nucleic Acids Res.

[92] Spandidos A, Wang X, Wang H, Seed B (2010). PrimerBank: a resource of human and mouse PCR primer pairs for gene expression detection and quantification. Nucleic Acids Res.

[93] Wang X, Seed B (2003). A PCR primer bank for quantitative gene expression analysis. Nucleic Acids Res.

[94] Zerbino DR (2018). Ensembl 2018. Nucleic Acids Res.

