# Supplementary material for: Integration of TGF-β-induced Smad signaling in the insulin-induced transcriptional response in endothelial cells
Source: Sci Rep. 2019 Nov 18;9:16992. doi: 10.1038/s41598-019-53490-x (PMC6861289; doi:10.1038/s41598-019-53490-x)
Supplement: Supplementary file 1 — Supplemental Figures [file 41598_2019_53490_MOESM1_ESM.pdf]

Integration of TGF-β-induced Smad signaling in the insulin-induced transcriptional response in endothelial cells

Erine H. Budi[1], Steven Hoffman[1], Shaojian Gao[2], Ying E. Zhang[3] and Rik Derynck[1]*

[1]Departments of Cell and Tissue Biology, and Anatomy, Eli and Edythe Broad Center of Regeneration Medicine and Stem Cell Research, University of California at San Francisco, San Francisco, CA 94143-0669, USA; [2]Thoracic and Gastrointestinal Oncology Branch, Center for Cancer Research, National Cancer Institute, Bethesda, MD 20892-1906, USA; [3]Laboratory of Cellular and Molecular Biology, Center for Cancer Research, National Cancer Institute, Bethesda, MD 20892-4256, USA.

SUPPORTING INFORMATION

Supplemental Figure S1. Original gel blots of HUVECs treated with insulin (Ins) in the presence or absence of SB431542 for 30 min using the indicated concentrations.

Supplemental Figure S2. Dose response of insulin-induced Smad activation in HMVEC-L cells.

Supplemental Figure S3. Original gel blots of HMVEC-L cells treated with insulin (Ins) in the presence or absence of SB431542 for 30 min using the indicated concentrations.

Supplemental Figure S4. Effects of anti-TGF-β antibody 1D11 on relative mRNA expression of selected genes in the absence or presence of insulin.

Supplemental Figure S5. Dose-dependent relative mRNA responses to insulin and/or SB431542.

Supplemental Figure S6. Relative mRNA expression of selected genes in HMVEC-L cells.

Supplemental Figure S7. Dose response of insulin on gene expression.in HMVEC-L cells.

Supplemental Figure S8. Relative mRNA expression in response to TGF-β..

Supplemental Table 1. List of genes that are differentially expressed after 90 min treatment.

Supplemental Table 2. List of genes that are differentially expressed after 6 h treatment.

Supplemental Table 3. Lists identified byVenn diagram analysis of common insulin- and SB431542- regulated genes.

Supplemental Table 4. KEGG pathway and Gene Ontology (GO) for biological process enrichment analysis of the 175 upregulated genes in response to insulin.

Supplemental Table 5. KEGG pathway and Gene Ontology (GO) for biological process enrichment analysis of the 41 downregulated genes in response to insulin.

Supplemental Table 6. KEGG pathway and Gene Ontology (GO) for biological process enrichment analysis of the 248 upregulated genes in response to SB431542.

Supplemental Table 7. KEGG pathway and Gene Ontology (GO) for biological process enrichment analysis of the 159 downregulated genes in response to SB431542.

Supplemental Table 8. Expression of common genes identified by Venn diagram analysis of insulin, SB431542, and insulin+ SB431542 group at 90 min treatment.

Supplemental Table 9. Expression of common genes identified by Venn diagram analysis of insulin, SB431542, and insulin+ SB431542 group at 6 h treatment.

Supplemental Table 10. List of common genes shared at 90 min and 6 h treatment.

Supplemental Table 11. List of the 89 and 370 genes and their expression levels presented in the log2 values.

Supplemental Table 12. Functional annotation enrichment for the 89 common genes identified at 90 min of treatment.

Supplemental Table 13. Functional annotation enrichment analysis for the 370 common genes identified at 6 h of treatment.

Supplemental Table 14. Functional annotation enrichment analysis for the 68 genes identified as common between 90 min and 6 h treatments.

Supplemental Table 15. Expression of the 68 genes identified as common between the 90 min and 6 h treatments.

Supplemental Table 16. Primers used for qRT-PCR analyses of genes.

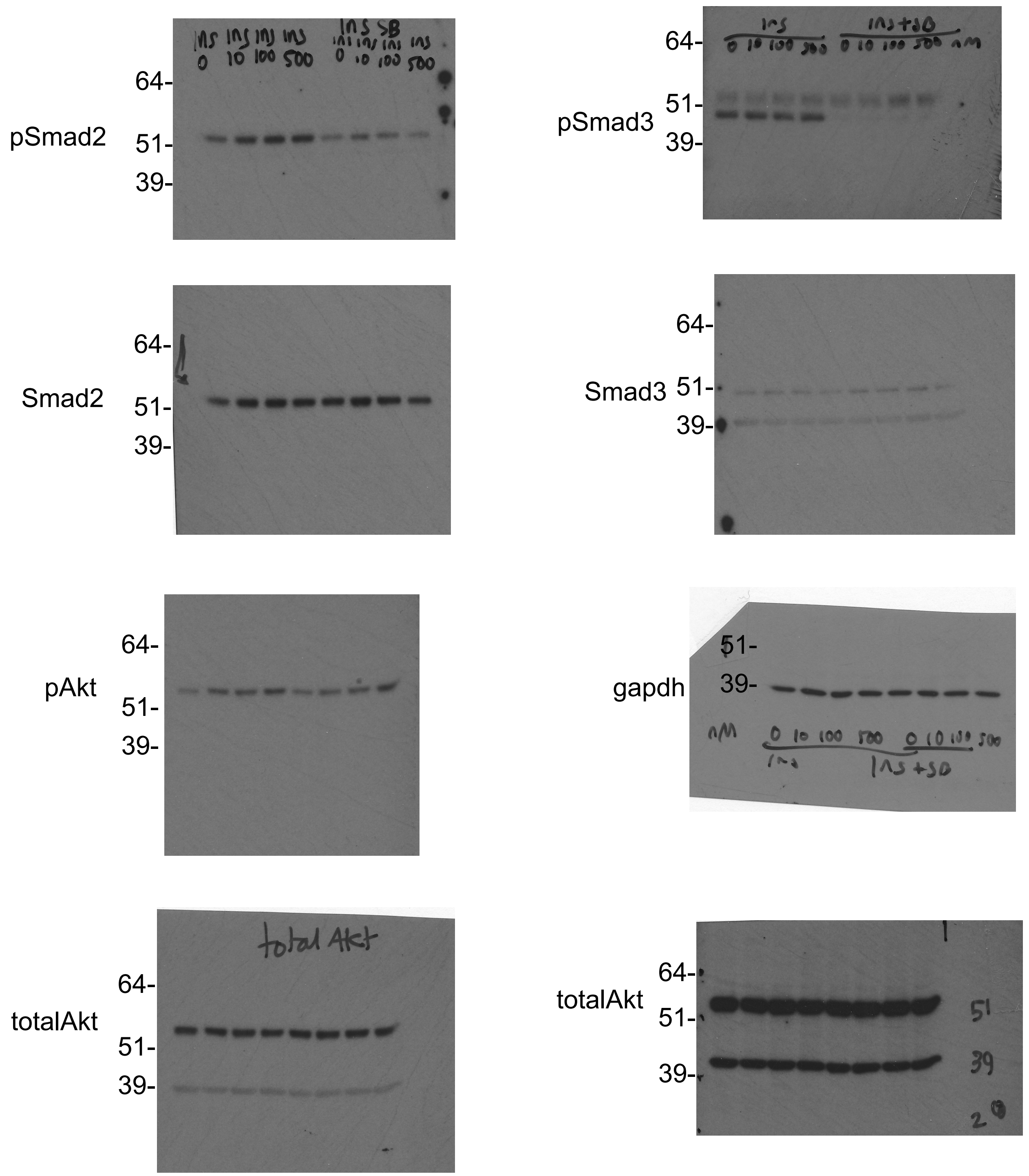

Supplemental Figure S1. Original gel blots of HUVECs treated with insulin (Ins) in the presence or absence of SB431542 for 30 min using the indicated concentrations. This figure is to accompany Fig 1.

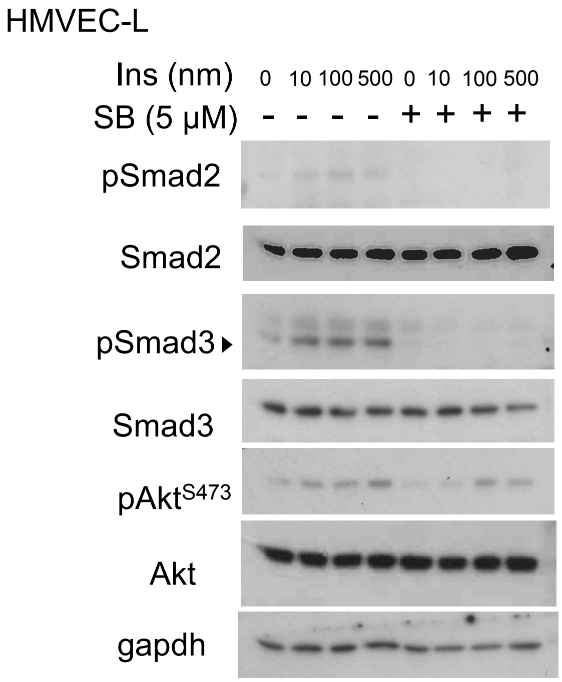

Supplemental Figure S2. Dose response of insulin-induced Smad activation in HMVEC-L cells. Cells were treated with insulin in the presence or absence of SB431542 for 30 min at the indicated concentrations. Smad2 and Smad3 activation were assessed by immunoblotting for phosphorylated Smad2 (pSmad2) or phosphorylated Smad3 (pSmad3). Insulin-induced Akt activation was assessed by immunoblotting for phosphorylated Akt (pAktS473). GAPDH was used as loading control. This figure is to accompany Fig 1.

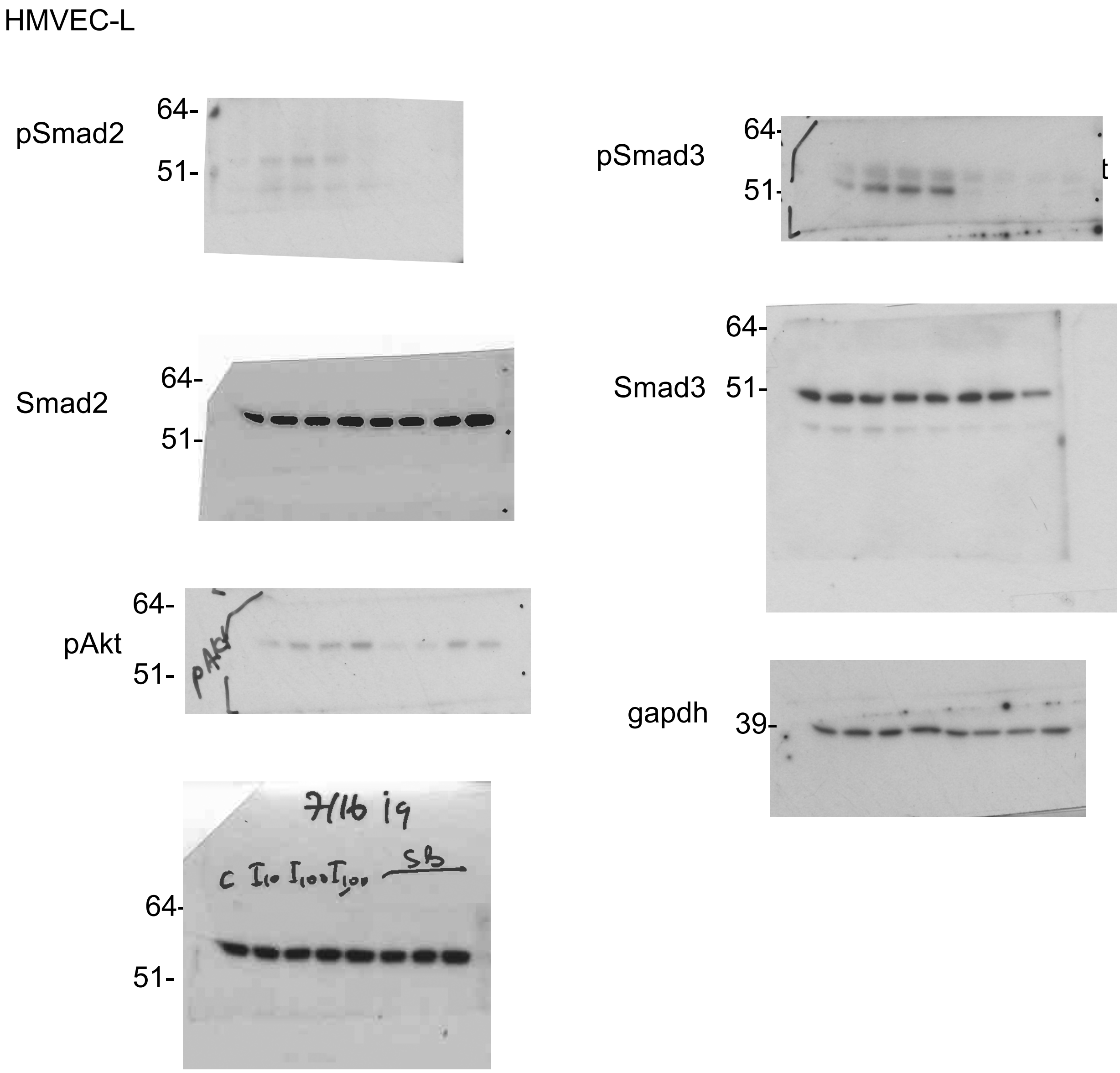

Supplemental Figure S3. Original gel blots of HMVEC-L cells treated with insulin (Ins) in the presence or absence of SB431542 for 30 min using the indicated concentrations. This figure is to accompany Fig S2.

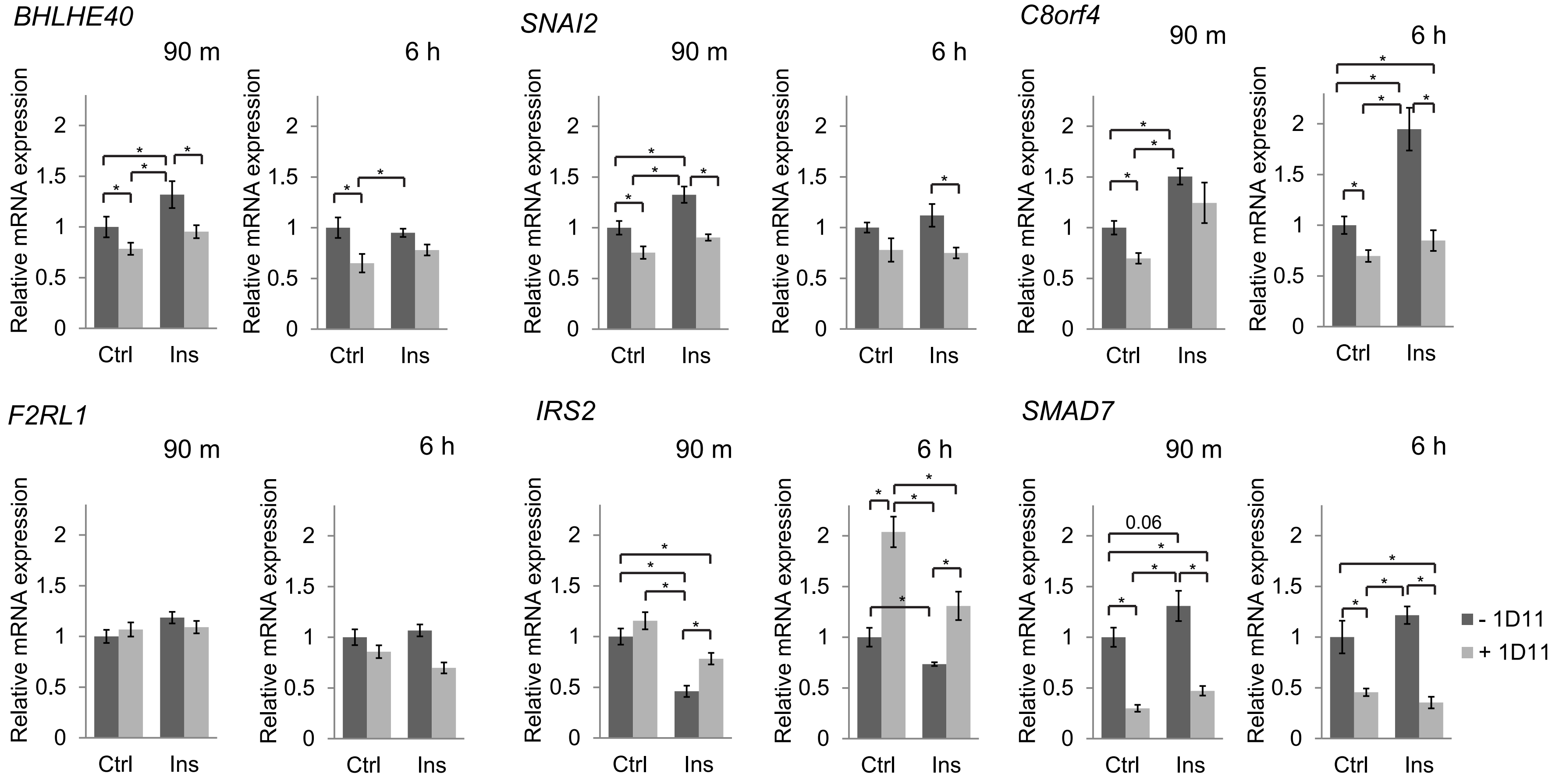

Supplemental Figure S4. Effects of anti-TGF-β antibody 1D11 on relative mRNA expression of selected genes in the absence or presence of insulin. HUVECs were treated with or without 100 nM insulin in the absence or presence of 1D11 for 90 min or 6 hours. mRNA expression was measured using qRT-PCR, and values were normalized to RPL13 mRNA. The statistical significance was determined by Wilcoxon test. Error bars indicate standard error of the means, based on three independent experiments. *p<0.05. This figure is to accompany Fig 6.

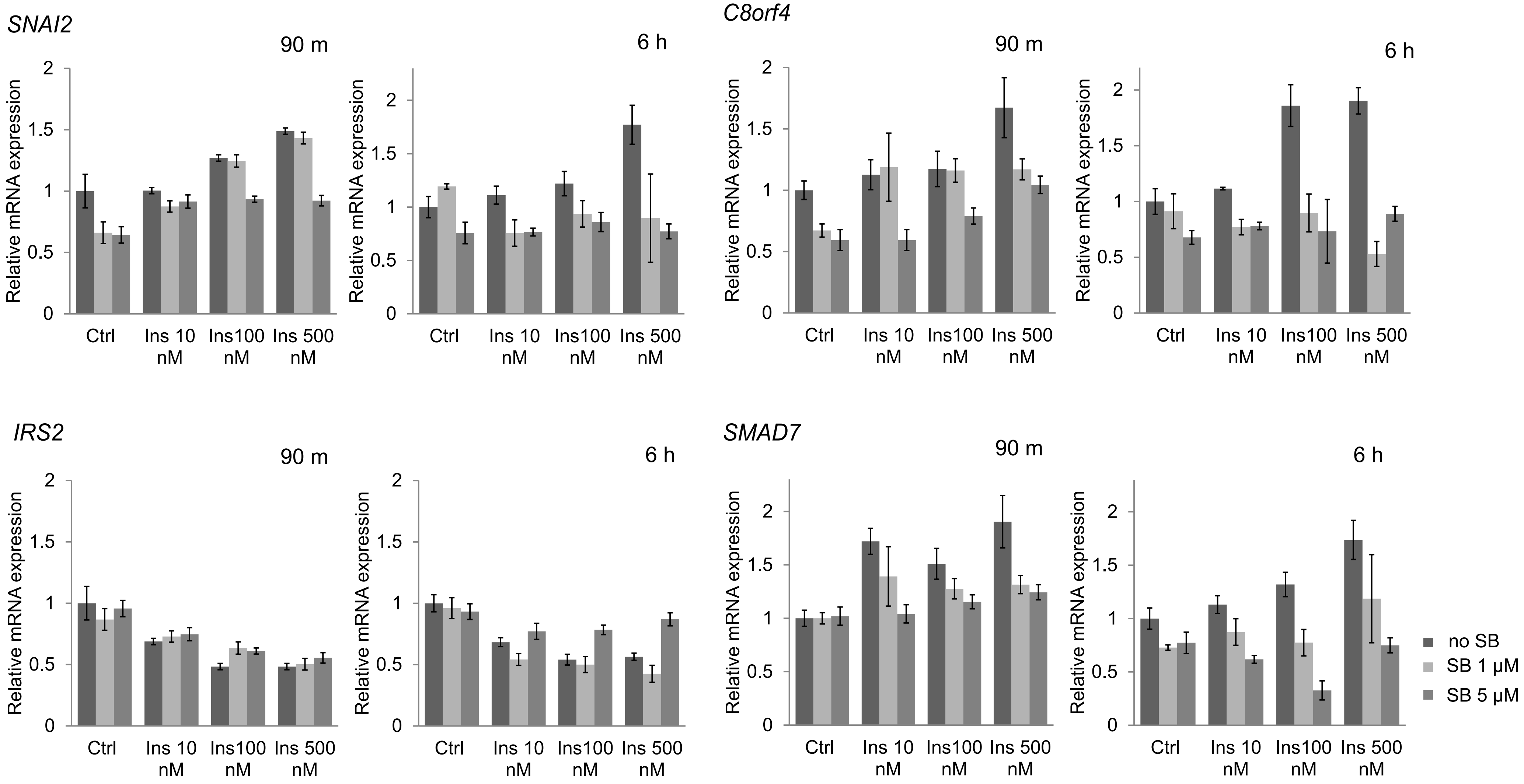

Supplemental Figure S5. Dose-dependent relative mRNA responses to insulin and/or SB431542. HUVECs were treated with or without insulin at the indicated concentrations in the presence or absence of 1 or 5 μM SB431542 for 90 min or 6h. mRNA expression of the indicated genes was measured using qRT-PCR, and values were normalized to RPL13 mRNA. Error bars indicate standard error of the means, based on three independent experiments. This figure is to accompany Fig 6.

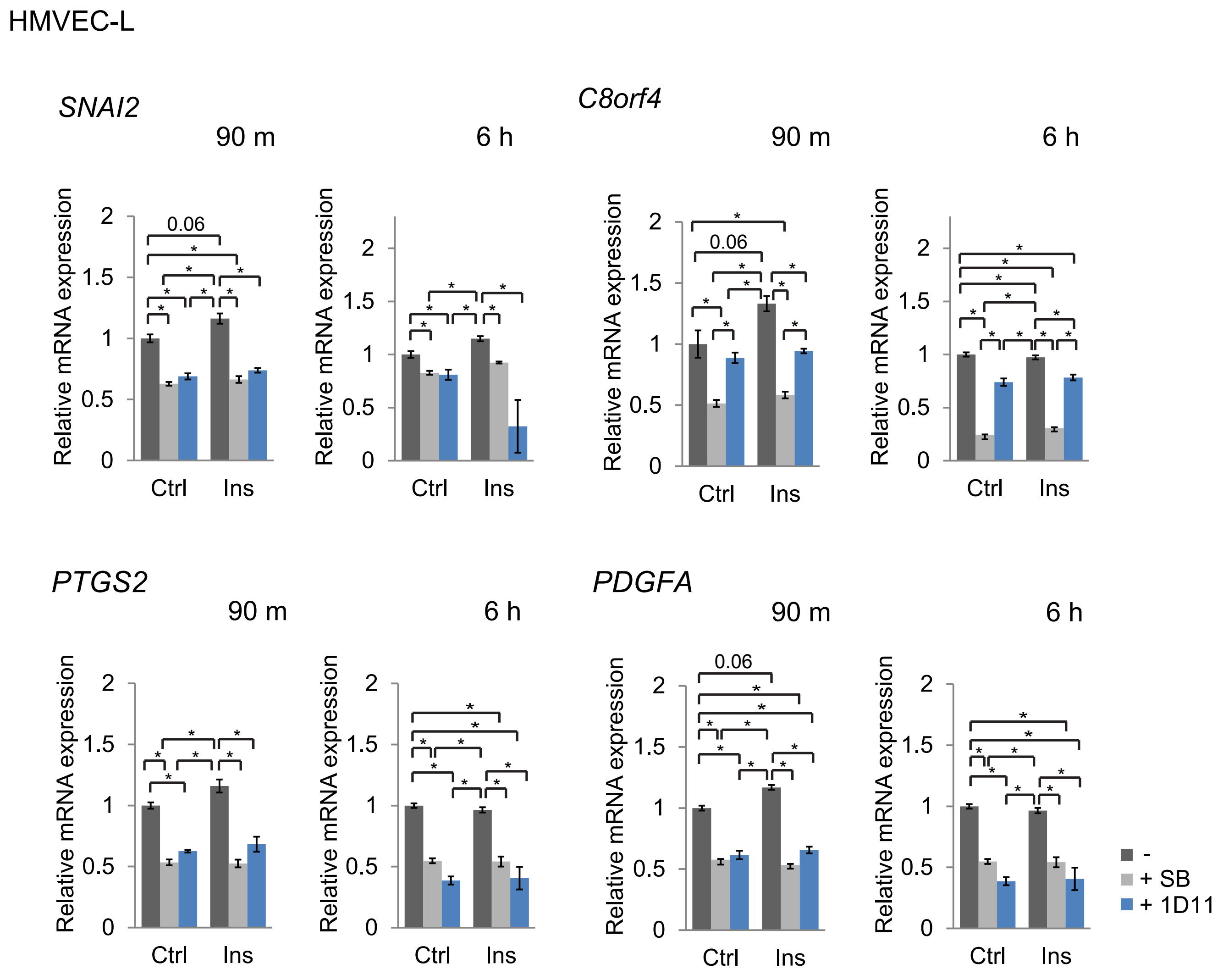

Supplemental Figure S6. Relative mRNA expression of selected genes in HMVEC-L cells. Cells were treated with or without 100 nM insulin in the presence or absence of TβRI kinase inhibitor SB431542 (SB) or anti-TGF-β antibody 1D11 for 90 min or 6 hours. mRNA expression was measured using qRT-PCR, and values were normalized to RPL13 mRNA. The statistical significance was determined by Wilcoxon test. Error bars indicate standard error of the means, based on three independent experiments. *$p<0.05$. This figure is to accompany Fig 6.

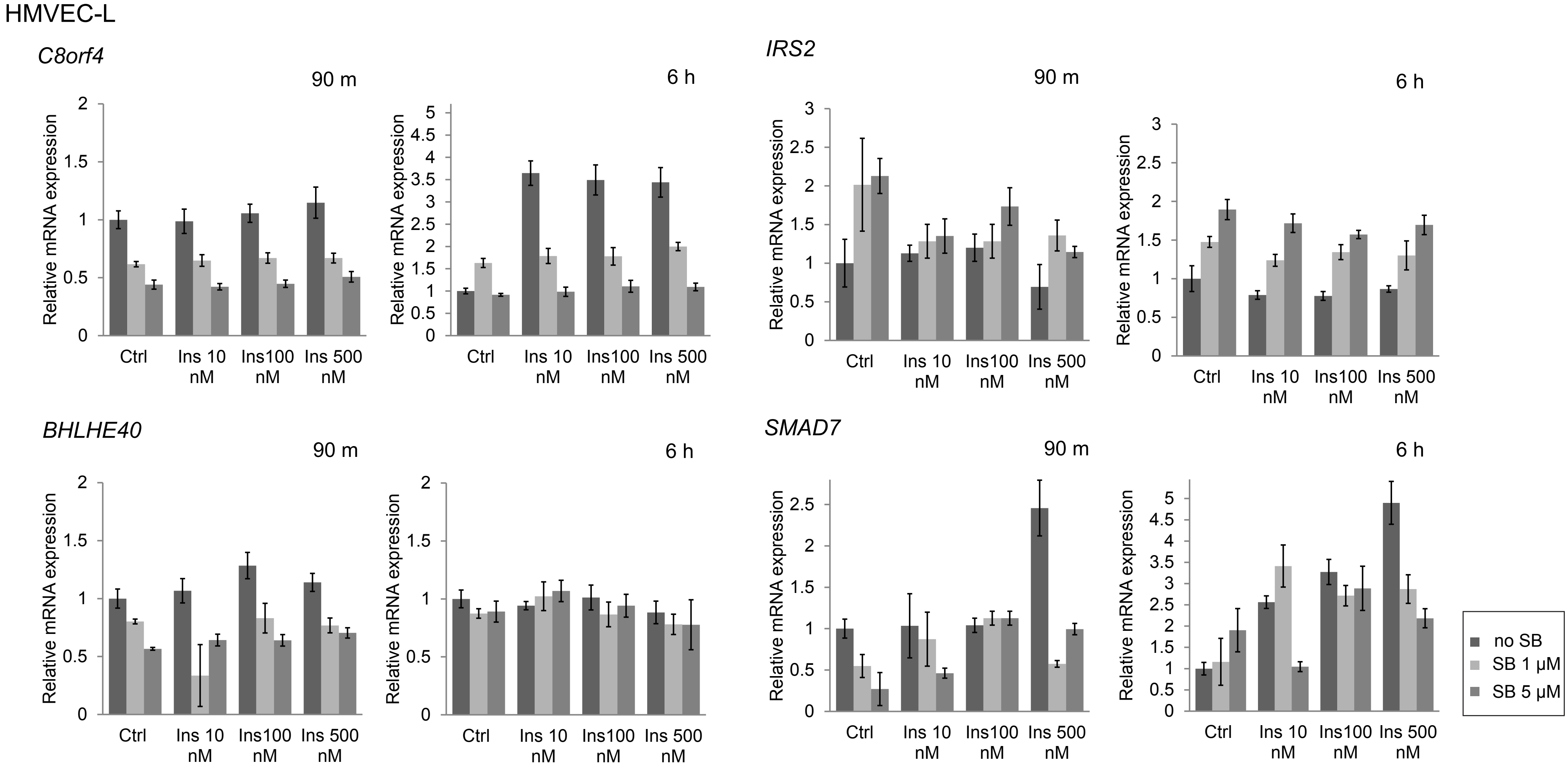

Supplemental Figure S7. Dose response of insulin on gene expression in HMVEC-L cells. Cells were treated with 10, 100, or 500 nM insulin in the presence or absence of 1 or 5 μM SB431542 for 90 min or 6 h. mRNA expression was measured using qRT-PCR, and values were normalized to RPL13 mRNA. Error bars indicate standard error of the means, based on three independent experiments. This figure is to accompany Fig 6.

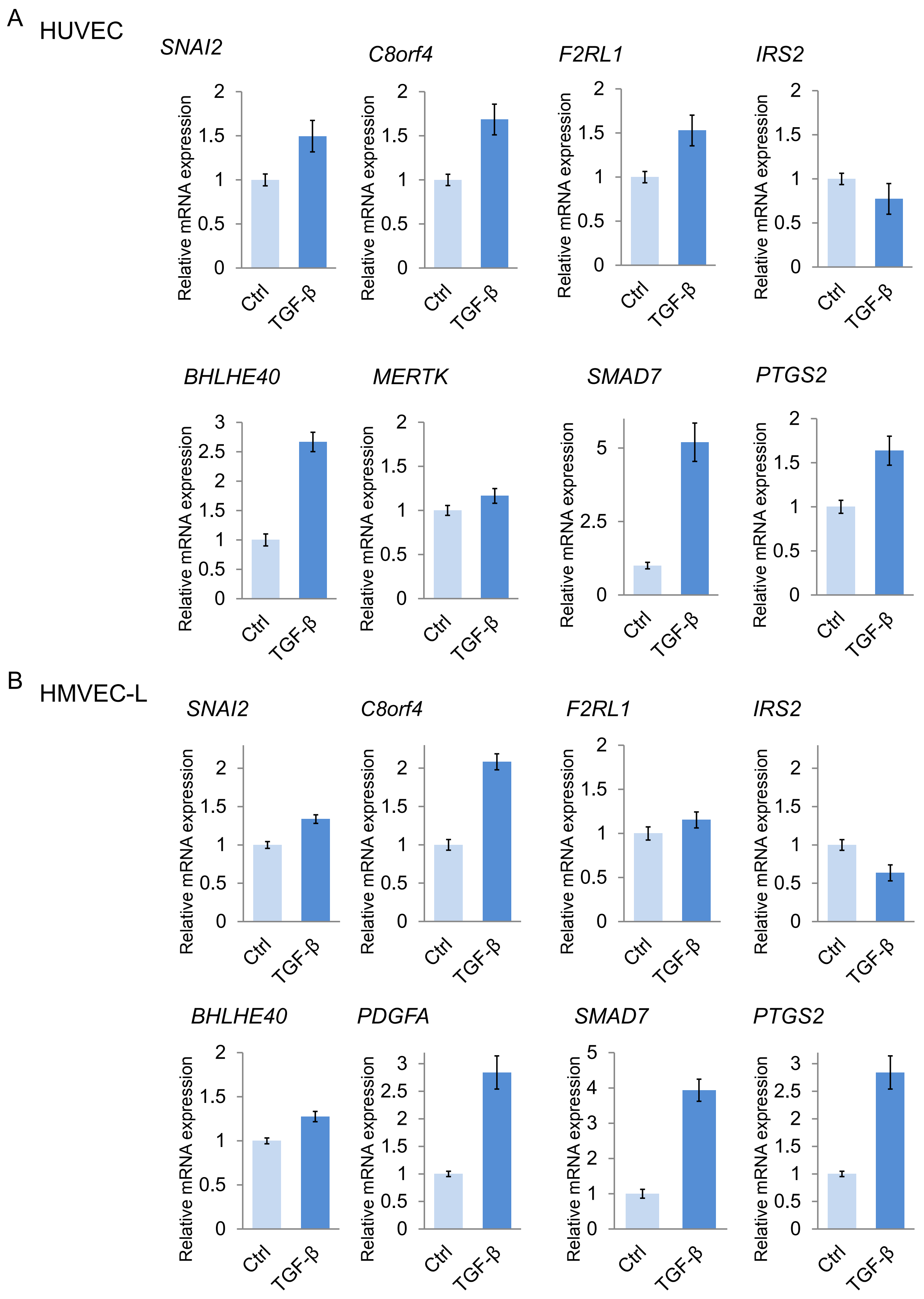

Supplemental Figure S8. Relative mRNA expression in response to TGF-ß quantified by qRT-PCR. HUVECs (A) and HMVEC-L cells (B) were treated with or without TGF-ß for 90 min. mRNA expression of the indicated genes was measured using qRT-PCR, and values were normalized to RPL13 mRNA levels. Error bars indicate standard error of the means. Representative figure is shown (n=2).This figure is to accompany Fig 8.
